# From gut to brain: understanding the role of microbiota in inflammatory bowel disease

**DOI:** 10.3389/fimmu.2024.1384270

**Published:** 2024-03-21

**Authors:** Siyu Wang, Shuwei Zhou, Zhongyu Han, Bin Yu, Yin Xu, Yumeng Lin, Yutong Chen, Zi Jin, Yalong Li, Qinhan Cao, Yunying Xu, Qiang Zhang, Yuan-Cheng Wang

**Affiliations:** ^1^ Zhongda Hospital, School of Medicine, Southeast University, Nanjing, China; ^2^ Department of Gastroenterology, The First Hospital of Hunan University of Chinese Medicine, Changsha, China; ^3^ Nurturing Center of Jiangsu Province for State Laboratory of AI Imaging & Interventional Radiology, Department of Radiology, Zhongda Hospital, School of Medicine, Southeast University, Nanjing, China; ^4^ Eye School of Chengdu University of Traditional Chinese Medicine, Chengdu, China; ^5^ The Second Clinical Medical College, Zhejiang Chinese Medical University, Hangzhou, China; ^6^ Department of Anesthesiology and Pain Rehabilitation, Shanghai YangZhi Rehabilitation Hospital (Shanghai Sunshine Rehabilitation Center), School of Medicine, Tongji University, Shanghai, China; ^7^ Anorectal Department, The Third Affiliated Hospital of Yunnan University of Chinese Medicine, Kunming, China; ^8^ School of Clinical Medicine, Chengdu University of Traditional Chinese Medicine (TCM), Chengdu, China; ^9^ Clinical Medical School, Affiliated Hospital of Chengdu University, Chengdu, China

**Keywords:** inflammatory bowel disease, psychological disorders, comorbidities of mind and body, brain-gut-microbiota axis, treatment strategy

## Abstract

With the proposal of the “biological-psychological-social” model, clinical decision-makers and researchers have paid more attention to the bidirectional interactive effects between psychological factors and diseases. The brain-gut-microbiota axis, as an important pathway for communication between the brain and the gut, plays an important role in the occurrence and development of inflammatory bowel disease. This article reviews the mechanism by which psychological disorders mediate inflammatory bowel disease by affecting the brain-gut-microbiota axis. Research progress on inflammatory bowel disease causing “comorbidities of mind and body” through the microbiota-gut-brain axis is also described. In addition, to meet the needs of individualized treatment, this article describes some nontraditional and easily overlooked treatment strategies that have led to new ideas for “psychosomatic treatment”.

## 1 Introduction

Inflammatory bowel disease (IBD) is a type of chronic recurrent inflammation of the intestine that involves abnormal immune responses. The main types of IBD are ulcerative colitis (UC) and Crohn’s disease (CD) (1). The causes of IBD onset are complex, with multiple factors such as nerves, the endocrine system, immunity, gut microbiota, and psychological factors, being involved (2). Since the beginning of the 21st century, IBD has become a global public health problem. According to statistical data (3), it is expected that by 2025, the number of IBD patients in China will reach 1.5 million. This estimate is consistent with years of research experience, that is, with socioeconomic development, the incidence rate of IBD will increase annually (4).

The pathogenesis of IBD has not yet been fully elucidated. Studies have shown that environmental factors and psychological stress may be key factors affecting the course of IBD (5, 6). Recently, the bidirectional relationship between psychological stress and inflammatory activity has received considerable attention (7). An increasing number of IBD patients have psychosomatic comorbidities, which is mainly reflected in the fact that acute and chronic psychological stress can increase the incidence of IBD, and depression and anxiety are easily comorbid during and after the disease, which seriously affects the quality of life (QOL) of patients (8). A systematic review showed that the prevalence of depression and anxiety in IBD patients was 15% and 20%, respectively (9). Meta-analysis shows that IBD patients have a higher prevalence of anxiety and depression symptoms, with about one-third of patients affected by anxiety symptoms and a quarter affected by depression symptoms. Additionally, the prevalence of anxiety or depression symptoms in active IBD patients is higher than that in non-active disease patients (10). Studies have shown that depressed patients with gastrointestinal symptoms are more likely to develop IBD in the future than individuals with gastrointestinal discomfort alone, and the severity of perceived stress and mental symptoms increases the risk of IBD recurrence (11, 12). The incidence of depression in patients was greater than that in the control population (without IBD) 5 years before diagnosis (8). Moreover, compared to healthy controls, IBD patients reported a greater incidence of depression and anxiety (13). A case-control study involving approximately 12,500 patients showed significantly higher levels of anxiety and depression compared to the general population, especially within 12 months after diagnosis of IBD (14). Despite this evidence, the “comorbidities of mind and body” have not been fully recognized, and extensive experimental and clinical research on the mechanism of stress-IBD interactions is still needed.

Recently published research has proposed the concept of the brain-gut-microbiota axis (BGMA) and its role in the progression of IBD (15). Research has confirmed that intestinal health is closely related to psychological disorders (16). The BGMA includes the neuroendocrine pathway dominated by the hypothalamic pituitary adrenal axis (HPA), the enteric nervous system (ENS), the autonomic nervous system (ANS), and the gut microbiota (17). The communication system between the brain and the gastrointestinal tract has become an actively researched pathway for understanding the relationship between psychological disorders and IBD.

## 2 The stress- brain-gut signaling pathway -IBD

### 2.1 Neuroendocrine system

**Figure 1:**
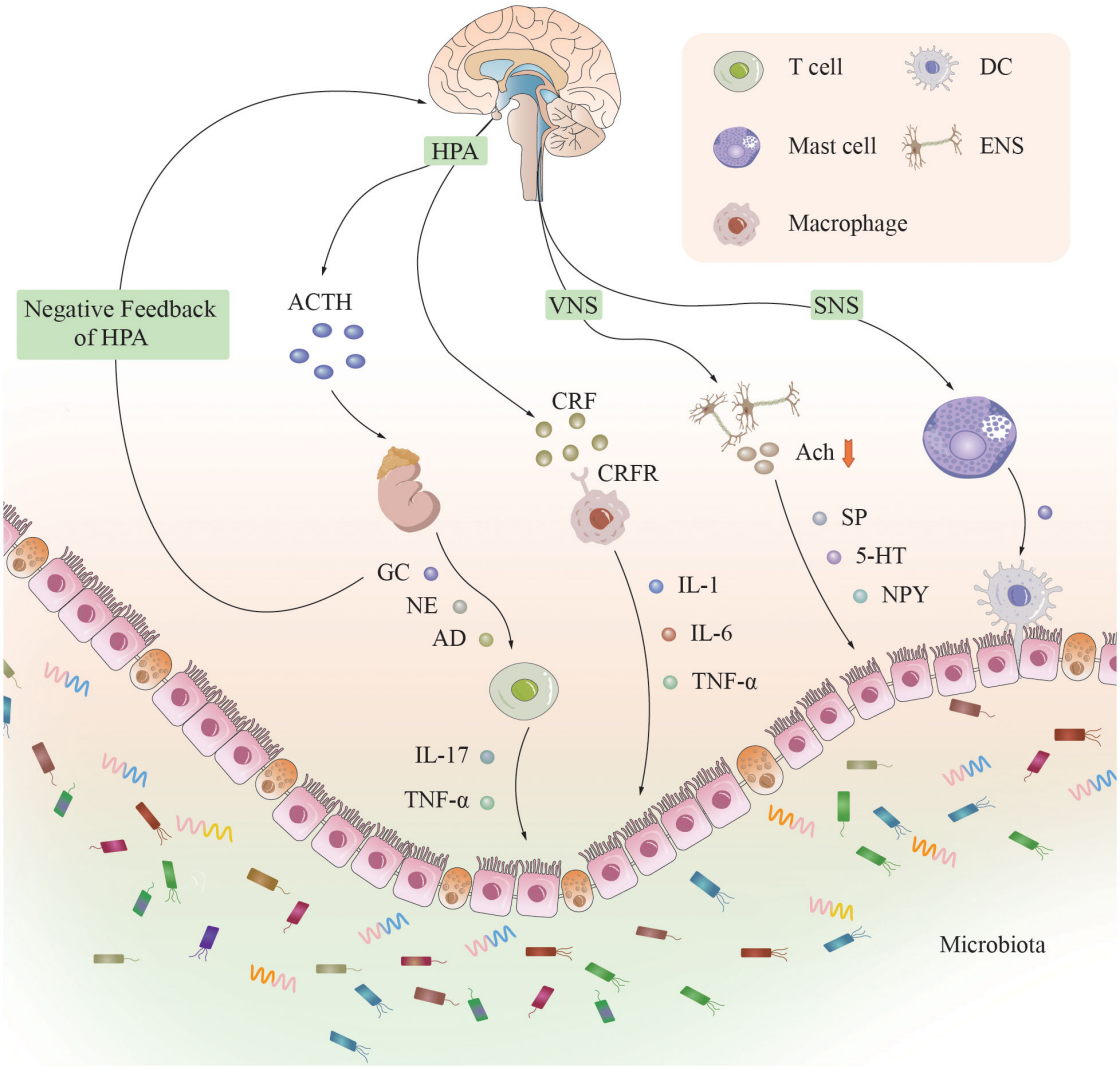
*The neural, endocrine, and immune systems in the brain-gut-microbiota axis.* A complex bidirectional communication network is constructed between the brain and the intestine through the brain-gut axis. The neuroendocrine system, immune system, and gut microbiota are its main components. When the brain senses stress, various systems and pathways are activated, affecting intestinal function by releasing neurotransmitters, inflammatory factors, and metabolites, and vice versa. [CRF]R, [corticotropin releasing factor] receptor; HPA, hypothalamic-pituitary-adrenal axis; ACTH, adrenocorticotropic hormone; GC, glucocorticoids; NE, norepinephrine; AD, adrenaline; [IL]-1, 6, 17, [interleukin]-1, 6, 17; TNF-α, tumor necrosis factor-α; VNS, vagal nervous system; SNS, sympathetic nervous system; Ach, acetylcholine; SP, substance P; 5-HT, 5-hydroxytryptamine; NPY, neuropeptide YY; ENS, enteric nervous system; DC, dendritic cell.

The BGMA is an important bridge that connects the inside and outside of the body, psychology, and physiology. When the body senses stress, the BGMA is activated, and its impact on the neuroendocrine system is mainly achieved through the HPA axis and the corticotropin releasing factor (CRF) signaling pathway (**Figure 1**) (18). Researchers believe that immune mediated inflammatory damage may be the key to the occurrence of UC, and CRF plays a core role in this process (19). CRF is found in both the intestine and the brain, and is released by macrophages, intestinal epithelial cells, and immune cells activated by gastrointestinal and peripheral pathways (20).

Under physiological conditions, adrenal cortical hormones secreted by the adrenal gland have a negative feedback inhibitory effect on the HPA axis. However, under chronic stress conditions, excessive secretion of CRF in the hypothalamus leads to increased secretion of adrenocorticotropic hormone (ACTH) in the pituitary gland, ultimately resulting in excessive secretion of adrenal cortical hormones and dysregulation of the negative feedback mechanism of the HPA axis. Approximately half of the patients with IBD (54%) with psychological disorders experienced their first depressive episode more than 2 years before the onset of IBD (21). The elevated concentrations of ACTH and cortisol in the serum of patients with severe depression, as well as the elevated cerebrospinal fluid levels of CRF, result in sustained hyperfunction of the HPA axis, significantly promoting colonic motility and increasing intestinal mucosal permeability, thereby allowing bacteria to cross the intestinal epithelial barrier to activate the mucosal immune response and migrate to secondary lymphatic organs (22), stimulating the innate immune system and exacerbating the intestinal inflammatory response (23). CRF can also exacerbate inflammatory reactions by promoting macrophage polarization (24). The increase in intestinal mucosal permeability caused by mast cell degranulation is also a key mechanism for promoting the intestinal inflammatory response (24). When CRF or mast cells are inhibited, the trend of a worsening intestinal inflammatory response will be reversed (25, 26). The spasm of gastrointestinal small vessels caused by the excitation of the HPA axis, the necrosis of the gastrointestinal mucosa, and the increased susceptibility of the body to exogenous stress factors caused by the hyperactivity of the HPA axis are all considered to be reasons for the increased incidence of IBD due to the abnormal function of the HPA axis (27).

Researchers have shown that in CRF1 receptor-deficient mice, the intestinal inflammatory response is significantly reduced, while in CRF2 receptor-deficient mice, the intestinal inflammatory response is significantly increased, suggesting that the CRF1 receptor can promote intestinal inflammation and that the CRF2 receptor can inhibit intestinal inflammation (28). The same research results also confirmed that the use of CRF1 antagonists can significantly increase vagus nerve (VN) activity and reduce sympathetic nerve (SN) activity, suggesting that the use of CRF1 antagonists is a potential method for treating IBD caused by ANS disorders (29). The neuroendocrine system mainly mediates the intestinal inflammatory response caused by chronic stress, and recent research has focused mainly on the HPA axis; however, this axis is affected by various factors and is closely linked to the ANS and various hormones. The mechanism of action is complex, and a single study cannot fully reflect its mechanism of action. Therefore, additional large-scale, multicenter human-based clinical studies are needed to elucidate the specific pathways involved.

For basic researchers, the neuroendocrine system presents a rich field for exploring the intricate interplay between stress and gastrointestinal health. The elucidation of the roles played by CRF and the HPA axis in modulating intestinal inflammation and immune responses lays a foundational framework for the identification of potential therapeutic targets that exert modulatory effects on intestinal inflammation, including CRF1 and CRF2 receptors. For clinicians, this knowledge holds substantial value. It underscores the potential for the development of novel therapeutic interventions for IBD and related disorders by targeting the CRF signaling pathway or modulating the HPA axis. Furthermore, the delineation of the relationship between psychological stress and the exacerbation of IBD symptoms highlights the imperative for integrative treatment modalities. Such modalities should not only tackle mental health concerns but also furnish more effective management strategies for patients suffering from stress-associated gastrointestinal conditions. This integrative approach could potentially ameliorate the prognosis of patients afflicted with conditions such as IBD, thereby underscoring the necessity for a comprehensive understanding of the neuroendocrine mechanisms underlying stress-related gastrointestinal pathologies.

### 2.2 ENS

The ENS refers to a neural system composed of primary sensory neurons, intermediate neurons, and motor neurons that regulate gastrointestinal effects in the gastrointestinal tract. The ENS is embedded within the intestinal wall and is interconnected with the intestinal endocrine system, immune system, peripheral nervous system (PNS), central nervous system (CNS), and gut microbiota. The ENS releases a large number of neurotransmitters and neuropeptides, including substance P (SP), 5-hydroxytryptamine (5-HT), somatostatin, vasoactive intestinal peptide (VIP), glutamate, peptide YY (PYY), etc. (**Table 1**), which affect the movement, immunity, and inflammatory response of the intestine (49, 50). Because of its great similarity to the brain, the ENS is also known as the second brain.

**Table 1:** The influence of neurotransmitters released by ENS on the brain-gut axis.

Neurotransmitters	Generation and expression	Mechanism
SP (30–34)	Multiple brain regions (including the hypothalamus, amygdala, lateral septum, and bed nucleus of the stria terminalis), intestinal smooth muscle, intestinal mucosa and submucosa, and various immune cells	Under stress, SP mainly binds to the non-peptide NK-1 receptor to promote intestinal inflammation. The production of inflammatory factors further increases the expression of NK-1 receptors. In addition, SP can also regulate the release of pro-inflammatory cytokines from adjacent mesenteric preadipocytes through IL-17, leading to UC.
5-HT (35–39)	5-HT is a monoamine molecule synthesized from tryptophan. Central 5-HT accounts for only a small portion of the total 5-HT in the body, and more than 90% of 5-HT is located in the gastrointestinal tract, mainly produced by enterochromaffin cells.	Inflammation can inhibit the activity of 5-HT transporters and alter the concentration of 5-HT in the mucosa, resulting in an increase in local 5-HT levels. After binding to SERT, 5-HT activates effector cells, leading to increased intestinal sensitivity, smooth muscle spasm, and damage to the intestinal physiological barrier, which can lead to UC.
Neuropeptide YY family (40–44)	NPY is expressed in multiple neuronal systems in the brain, from the medulla oblongata to the cerebral cortex, and is also expressed in enteric neurons, including secretory motor neurons and inhibitory motor neurons. PYY and PP are located in endocrine cells in the ileum, colon, and rectum.	There is a mutual influence between NPY in the brain and serotonin and somatostatin in the serum. NPY can reduce the concentration of serotonin and its metabolite 5-HIAA, and promote the inhibition of T lymphocyte proliferation by somatostatin and reduce the release of pro-inflammatory cytokines such as INF-γ.
Glutamate (45–48)	Under stress, the intestinal microenvironment changes, and enteric endocrine cells secrete glutamate as a neurotransmitter to transmit information to the brain.	Glutamate can lead to the formation of ROS through the activation of NMDA receptors and subsequent elevation of intracellular free Ca2^+^, as well as the production of NO through the activation of NOS. In addition, enteric glutamate can induce the expression of IL-1β and TNF-α in neuronal cells through NMDA receptors and regulate IL-1β-induced neuronal damage in vitro.

Elucidating the role of the ENS and its associated neurotransmitter release within the BGMA is imperative for a comprehensive understanding of the pathophysiological mechanisms underpinning gastrointestinal disorders, as well as their interplay with neural and psychosocial determinants. Recent investigations in this domain have catalyzed the exploration of novel therapeutic avenues, specifically through the identification of targeted molecular interventions. For instance, elucidation of SP and its interaction with the NK-1 receptor delineates a promising therapeutic target for pharmacological intervention in UC. Concurrently, the characterization of neurotransmitter expression profiles in UC presents the potential for the development of innovative biomarkers. These biomarkers could revolutionize diagnostic processes, prognostication, and the monitoring of therapeutic responses in UC management.

Existing therapeutic modalities, such as surgical interventions and systemic immunosuppression, offer a broad-spectrum approach with variable efficacy and significant adverse effects. In contrast, the advancement of therapies targeting specific neurotransmitter pathways heralds a new era of precision medicine. Such targeted approaches not only hold the promise of enhancing therapeutic outcomes but also aim to significantly ameliorate the QOL for patients afflicted with gastrointestinal diseases. This paradigm shift towards neurotransmitter pathway-specific interventions underscores a pivotal moment in the evolution of gastrointestinal disease management, emphasizing the need for continued research and development in this dynamic field of medical science.

### 2.3 ANS

The normal physiological function of the intestine is mainly controlled by the ANS, and ANS dysfunction manifests as increased sympathetic function and decreased vagal function, which may induce the production of inflammatory factors in the intestine of IBD patients (51). MAULE reported that in patients with UC, sympathetic tone increases at rest, whereas parasympathetic tone decreases, suggesting that the activation of the sympathetic nervous system (SNS) seems to be related to intestinal disease activity (52). The activated SNS can promote catecholamine release, cause mast cell degranulation, promote the secretion of inflammatory cytokines, increase intestinal mucosal permeability, and thus promote intestinal inflammatory reactions (53). Kyösola compared the results of immunofluorescence histochemistry of rectal mucosa between UC patients and healthy individuals and found that the number of SN fibers in the inflamed area of UC patients was significantly increased (54). After inhibiting SN fibers with the sympathetic blocker clonidine, the patients’ conditions significantly improved, and their disease activity indices also decreased. However, Straub compared the number of SN fibers in the intestinal mucosa and submucosa of DSS-induced chronic enteritis mice and healthy mice and found that the number of SN fibers in DSS-induced model mice was significantly decreased (55).

The above studies suggest that SNs can regulate intestinal inflammation through multiple pathways and that the role of SNs in intestinal inflammation may be completely different. Further in-depth study of the specific mechanism is needed.

VN is a major component of the parasympathetic nervous system, which is a mixed nerve with anti-inflammatory properties through its afferent and efferent fibers, located at the junction of the brain-gut axis (56). Research has shown that stress can lead to a decrease in heart rate variability in the human body, which indicates a decrease in VN tone. Stimulating VN to increase VN tone exhibits anti-inflammatory effects in the intestine. Therefore, impaired VN tone in response to stress may lead to an increased proinflammatory state and systemic inflammatory response in the intestine (57, 58). Animal experiments have shown that VN signaling can reduce intestinal inflammation by regulating the feeding behavior of mice and releasing acetylcholine (Ach) (59). Therefore, improving the function of the VN in patients may help treat UC. VN signaling is believed to have anti-inflammatory effects, and stress reduces the outflow of the VN and increases the outflow of the SN and the activity of the adrenal medulla, leading to an increase in norepinephrine and epinephrine levels (60). A decrease in the outflow of the VN and an increase in sympathetic tone can lead to the suppression of immune cell function and ultimately to intestinal inflammation. In addition, researchers have found in animal models of infectious shock that stimulating VN terminals can inhibit the occurrence and development of shock (61). Subsequent studies identified Ach as the mediator released by VN terminals, and further research revealed that Ach inhibits the release of shock effectors (IL-1 and TNF-α) by binding to α7-nicotinic-acetylcholine receptors (α-7-nAchRs) on macrophages. TNF-α is considered to be a key inflammatory mediator involved in IBD and remains the most widely used and effective current biotherapy target in IBD. A positive correlation has been found between vagotomy and the subsequent development of IBD, especially for CD, highlighting the importance of the integrity of the VN in preventing IBD (62). VN signaling has this anti-inflammatory effect through the close association of this nerve with splenic SN fibers in the celiac ganglion (63) or through nonneuronal communication pathways that have not yet been characterized. VN signaling increases catecholamine signaling to the spleen and induces splenic T cells to secrete Ach (64). These Ach-releasing T cells represent a new key finding that reveals an important mechanism of the cholinergic anti-inflammatory pathway. Similar T cells can also be found in lymph nodes and Peyer’s patches in mice and are similarly regulated by noradrenergic neurons (64). The Ach released by these T cells acts on circulating and tissue cells via α-7-nAchRs, thereby completing the cholinergic anti-inflammatory pathway. In addition, due to its central role, persistent inflammation can lead to psychological disorders, which in turn may cause flare-ups of the disease.

These research findings provide new therapeutic targets and methods, particularly in the search for nontraditional, neuromodulatory based approaches for treating IBD. For example, by activating the cholinergic anti-inflammatory pathway of VN signaling or using α-7-nAchRs agonists as a treatment option may bring new hope for IBD patients, especially for those who do not respond well to traditional treatment strategies.

### 2.4 Gut microbiota

Psychological stress is associated with a disruption of intestinal physiology, and this association has been supported by human and animal model studies (65, 66). The gut microbiome is considered to be the third key component of the brain-gut axis, and the concept of the microbiome-gut-brain axis (MGBA) has been established (67, 68). Psychological stressors can alter the composition of the gut microbiota and promote intestinal inflammation (69, 70).

Stress-induced dysbiosis is characterized by a decrease in the abundance of lactic acid bacteria, accompanied by an increase in bacterial translocation and a decrease in the host’s ability to resist pathogen infection (71). Studies have shown that social psychological stress can induce significant changes in the gut microbiota of mice and can increase the expression levels of proinflammatory factors such as interleukin-6 (IL-6) in peripheral blood (70). Antibiotics can eliminate this change, suggesting that gut dysbiosis is a prerequisite for the development of a systemic inflammatory response. Mice subjected to chronic social failure stress exhibit a decrease in the abundance and functional diversity of the gut microbiota, specifically a significant decrease in Bacteroides and a significant increase in Clostridium in the gut microbiota. At the same time, the abundance of genes involved in the synthesis and metabolism of neurotransmitter precursors and short-chain fatty acids (SCFAs) decreases (72). By establishing a chronic unpredictable mild stress (CUMS) mouse model, researchers found that the abundance of Lactobacillus in the gut microbiota significantly decreased. CUMS leads to a decrease in the diversity of the gut microbiota, with a significant increase in pathogenic bacteria (such as Escherichia and Shigella) and conditional pathogens (such as Enterococcus, Vagococcus and Aerococcus) (73). By establishing a model of chronic social stress in mice (70), researchers found that the depressive-like behavior of mice may be related to certain strains of bacteria at the species level, including *Clostridium leptum*, *Blautia coccoides*, and *Streptococcus hyointestinalis* (74). In a cohort of mice exposed to a single stressor, compared to that in a conventional home cage control setting, the expression of serum lipopolysaccharide-binding protein was increased, indicating that bacterial translocation occurs in parallel with colonic mucus disruption (75).

Some studies have shown that certain bacterial species in the gut microbiota can regulate the differentiation of intestinal T cells and play an indispensable role in the development and maintenance of the intestinal immune system (76). For example, segmented filamentous bacteria (SFB) induce the differentiation of T helper 17 (Th17) cells through major histocompatibility complex class II (MHCII)-dependent antigen presentation by intestinal dendritic cells (DCs) and innate lymphoid cells (ILCs), while polysaccharide A (PSA) derived from *Bacteroides fragilis* can activate Toll-like receptor (TLR) 2 on plasmacytoid DCs, thereby inducing the differentiation of regulatory T (Treg) cells (77). In mice subjected to chronic subordinate colony housing (CSC) preconditioning, the abundance of Helicobacter and Paraprevotella was significantly increased and the host immune response was activated. After the addition of DSS, the number of mesenteric lymph node cells in the mice further increased, the cells produced more IFN-γ and IL-6, and the intestinal tissue damage worsened (78). Similarly, male C57BL/6 mice subjected to chronic restraint stress (CRS) preconditioning and subsequent DSS intervention showed a more significant increase in the abundance of proinflammatory bacteria, such as Helicobacter and Streptococcus, which activate inflammatory signaling pathways such as the IL-6/STAT3 pathway, leading to colitis.

Stress induced changes in gut microbiota, characterized by a decrease in beneficial bacteria and increased susceptibility to pathogens, are associated with changes in systemic inflammatory response, neurotransmitters, and SCFAs synthesis. Future research can explore interventions targeting the gut microbiota to alleviate the effects of stress, such as probiotics or dietary adjustments, and their efficacy in preventing or reversing stress-induced changes in gut health.

## 3 The gut–brain signaling pathway and psychological disorders in IBD

The possible pathophysiological mechanism of brain-gut pathway activation after psychological stress, which leads to gastrointestinal diseases such as IBD, has been described in detail previously. At the same time, there is also significant evidence that IBD can cause or exacerbate psychological disorders through the gut-brain pathway (79, 80). The presence of psychological disorders not only causes increased gastrointestinal symptoms in IBD patients, but also may have other harmful consequences, including decreased compliance with medication treatment, repeated consultations for the disease, and increased clinic visits (81).

### 3.1 The role of the VN

Existing research indicates that the intestinal immune system can affect the brain through multiple pathways (82). One mechanism is through the VN afferent signaling pathway. Before lipopolysaccharide (LPS) induces peripheral inflammation in rodents, cutting the VN can prevent certain disease behaviors, including fatigue, social withdrawal, cognitive dysfunction, and psychological changes (mainly for depression, as the improvements in symptoms of anxiety are still questionable) (83). The afferent fibers of the VN are sensitive to changes in the gut environment, including the presence of inflammation. These fibers can detect proinflammatory cytokines and other inflammatory markers produced in the gut. Once these inflammatory signals are detected, the VN transmits this information to the brainstem, specifically to the nucleus tractus solitarius (NTS), which then relays information to higher brain centers involved in mood regulation, such as the amygdala, hippocampus, and prefrontal cortex (84). Additionally, early studies have shown that the VN is involved in the transmission of peripheral-derived IL-1β in the CNS during fever induction (85), and this transmission process is eliminated in rats subjected to vagotomy.

From another perspective, the VN fibers are distributed throughout all layers of the digestive wall, but do not pass through the epithelial layer, so they do not come into direct contact with the gut microbiota (86). However, due to its location and various receptors, including mechanical and chemical receptors, the VN can detect and respond to various signals, including metabolites of the gut microbiota, gut hormones or neurotransmitters (87). VN chemoreceptors can participate in communication between the gut microbiota and the brain by detecting SCFAs. Its mechanism is related to the fact that SCFAs can bind to the G-protein coupled receptors carried by enteroendocrine cells, thereby stimulating VN (88). There is evidence to suggest that injecting *Lactobacillus johnsonii* into the duodenum can increase VN activity (89). Chronic *Lactobacillus rhamnosus* (*L. rhamnosus*) treatment induced changes in gamma-aminobutyric acid (GABA) brain expression in healthy mice. These mice also exhibited a decrease in cortisol levels associated with stress and behaviors (anxiety and depression). After vagotomy, these effects were not observed (90). Fecal microbiota transplantation (FMT) from healthy donors to psychologically stressed mice can improve brain dysfunction, and the degree of benefit from this improvement is significantly reduced after vagotomy, indicating that some beneficial effects of FMT may be mediated by the VN (91). The above evidence once again indicates that the VN is the main regulatory structure of the gut-brain axis.

Further research on the mechanism of VN, particularly how it detects and responds to signals from the gut, including proinflammatory cytokines and metabolites of the gut microbiota (such as SCFAs), helps to understand the complex interactions between the gut microbiota and the brain, providing valuable models for understanding how peripheral signals affect mental health.

### 3.2 The hippocampus and psychological disorders

Circulating cytokines released by periventricular organs and leukocyte infiltration into the brain, as well as TLR activation on macrophages in periventricular organs, lead to the production of proinflammatory cytokines and their diffusion into the brain (83). An analysis of the relationship between the levels of proinflammatory cytokines and brain region changes in a mouse model of colitis, revealed that an increase in circulating proinflammatory cytokine levels affected several brain regions, most importantly the hippocampus (92, 93). The hippocampus is part of the limbic system in the brain that controls psychological health. In a study of dinitrobenzene sulfonic acid (DNBS)-induced colitis model mice, it was found that the mice exhibited behaviors consistent with depression and anxiety (92). Moreover, in an examination of the brains of these mice, and it was found that the expression of genes related to inflammation and excessive NO production was increased in the hippocampus. The same research results confirmed that in the hippocampus of colitis model mice, the TNF and NO concentrations and inducible NOS expression were increased (93). In addition, intraperitoneal injection of NOS inhibitors reduced the concentrations of TNF and NO in the hippocampus, reversing the anxiety- and depression-like behaviors, although this seemed to have little effect on the improvement of colitis. The increase in proinflammatory cytokine levels in the hippocampus may be related to a reduction in hippocampal neurogenesis. For example, 2,4,6-trinitrobenzenesulfonic acid solution (TNBS)-induced colitis was shown to reduce the synaptic plasticity of glutamatergic neurons in the hippocampus and increase synaptic transmission (94).

Notably, neuroinflammation can have a negative impact on adult hippocampal neurogenesis. Although homeostatic microglia support adult hippocampal neurogenesis, peripheral inflammatory factors such as TNF and IL-1β inhibit the proliferation and maturation of neural precursor cells (NPCs) (95). Although the direct mechanism linking neuroinflammation and adult hippocampal neurogenesis in IBD has not been established, studies on acute and chronic DSS-induced colitis model animals have shown impaired adult hippocampal neurogenesis (96, 97). In summary, adult hippocampal neurogenesis is vulnerable to peripheral and neural inflammation, leading to neuropsychiatric symptoms associated with IBD. In another group of colitis model mice, inflammation led to an increase in the level of the p21 protein in the hippocampus (98). This protein can prevent the proliferation of early neuronal progenitor cells.

In recent years, the relationship between the gut microbiota and the hippocampus has received increasing attention from scholars. Probiotics, especially Lactobacillus strains, can significantly reduce serum CORT and ACTH levels and ameliorate hippocampus-dependent emotional changes (99, 100). Bifidobacterium strains can also reduce serum CORT levels and normalize anxiety behavior (101, 102). Studies have shown that FMT can significantly improve depression-related performance and the composition of the fecal microbiota after CUMS, reverse increases in serum IL-6 and TNF levels, decrease 5-HT levels, and decrease hippocampal GABA levels (103).

There is a clear pathway to further investigate the detailed mechanisms by which intestinal inflammation leads to changes in brain chemistry and structure, with a particular focus on the pathways that lead to elevated levels of brain cytokines and their direct impact on the hippocampus. The next step for researchers can explore the specific effects of inflammation on hippocampal neurogenesis and synaptic plasticity, investigate how different inflammatory markers affect neural precursor cells and synaptic transmission, and the positive role of probiotics or FMT in reducing neuroinflammation.

### 3.3 Important effects of neuroinflammation

In IBD patients, the immune system responds to gut bacteria in an exaggerated manner (104). Studies have shown that the gut microbiota interacts with the intestinal epithelium, inducing responses from T cells in the gut and microbial-specific T cells in the thymus (105, 106). In addition, as metabolic products derived from gut microbes, SCFAs provide energy support for local immune responses. SFCAs regulate the development of B cells and the differentiation of Treg cells, and participate in the activation of inflammatory bodies (107). Fecal concentrations of SCFAs in IBD patients are significantly reduced (108). In addition to SCFAs, the imbalance of gut microbes also affects bile acid and tryptophan metabolism, further increasing intestinal inflammation (107). In IBD patients and DSS-induced colitis models, bacterial translocation and increased levels of circulating inflammatory mediators confirm the gradual transition from local inflammation in the gastrointestinal tract to systemic inflammation (109). Once gut-derived peripheral inflammation spreads to the CNS, resident immune cells, such as astrocytes, microglia, and brain-resident macrophages, may be activated and enter an inflammatory state, where they produce various cytokines and chemokines (110). These immune factors and chemokines attract immune cells from the peripheral bloodstream into the brain, triggering neuroinflammation.

It has been reported that in a model of depression, anxiety, and inflammation induced by intraperitoneal injection of LPS during CUMS, microglia can mediate behavioral deficits (111, 112). The main pathways involved include microglia-astrocyte crosstalk via glutaminase-1 and silent information regulator 2 homolog 1 (Sirt1)-nuclear factor erythroid 2-related factor 2 (Nrf2)-hemoxygenase 1 (Ho-1) signaling through the clearance of reactive oxygen species (ROS) (113, 114). In addition, microglia can phagocytose and prune synapses. Microglial synaptic pruning is crucial for normal brain development, but it is abnormally upregulated during neurodegenerative diseases (115). Notably, that the complement system plays a key role in synaptic pruning and is associated with stress-induced depressive symptoms (116). In addition, in depression models, the interaction of microglia with synapses shows spatial and temporal differences. Early neuroinflammation (such as LPS-induced inflammation) exacerbates adolescent depressive-like behavior, which is associated with the formation of glutamatergic neurons spines in the anterior cingulate cortex (112). In summary, neuroinflammation in IBD can contribute to psychiatric symptoms by inducing structural changes in or the degradation of synapses or the death of neurons.

Overall, developing therapies that regulate the composition or metabolic output of gut microbiota (such as increasing SCFAs levels) and regulating the interaction between the immune system and gut microbiota may prevent cascade reactions that lead to systemic and CNS inflammation, reducing the likelihood of psychological disorders in IBD patients. Meanwhile, studying how neuroinflammation leads to psychological symptoms in IBD patients can guide the development of treatment methods targeting specific pathways, such as microglial astrocyte interactions or synaptic pruning mechanisms. The dynamic changes between intestinal inflammation, immune response, neuroinflammation, and psychological disorders have not been reported yet. By tracking the changes in these reactions over time in IBD patients, we can better understand the progression and interaction of these factors.

### 3.4 Brain-derived neurotrophic factor

Another key factor affecting psychological disorders in IBD patients is brain derived neurotrophic factor (BDNF). BDNF has a positive effect on the release of neurotransmitters and the expression and function of neurotransmitter receptors and ion channels (117). Moreover, BDNF can increase synaptic plasticity and adult neurogenesis. It has been reported that depression is associated with lower levels of BDNF (118). Notably, neuroinflammation reduces BDNF signaling, and BDNF expression is inhibited by IL-1β (119). The same study also confirmed that the BDNF levels in the brains of DSS-induced colitis model animals were significantly decreased (120, 121). Although neuroinflammation and a decrease in BDNF levels are synchronous in IBD models, no further research on whether there is a causal relationship is available. Therefore, this finding provides us with a direction for further research; that is, impaired BDNF signaling may be triggered by neuroinflammation, leading to psychiatric symptoms in individuals with IBD. In addition, BDNF can serve as a biomarker to identify IBD patients at higher risk of psychological disorders for early intervention.

### 3.5 The impact of the gut microbiota on psychological disorders in IBD patients

Changes in the gut microbiota may be both a cause and a consequence of intestinal inflammation and regulate neuropsychiatric symptoms by affecting the CNS while also having a direct effect on neurons. Therefore, exploring the changes in the CNS during IBD-related dysbiosis is particularly important for obtaining a better understanding of the pathogenesis of “comorbidities of mind and body”.

Different microbial metabolites can regulate the maturation and function of microglia (122). Microbial-derived SCFAs signaling via free fatty acid receptor 2 (FFAR 2) involves microglia, with its main component acetate being a key regulator of microglial metabolism and phagocytosis (122). Moreover, another component of SCFAs, propionate, can induce Treg cell activation and reduce the response of Th1 and Th17 cells (123). In addition, the bacterial metabolite tryptophan signals through the aryl hydrocarbon receptor to reduce the activation of astrocytes caused by microglial inflammation (124). Studies have shown that UC patients with depression or anxiety exhibit a unique gut bacterial profile associated with reduced blood levels of the metabolites 2′-deoxy-D-ribose and L-pipecolic acid (110). Interestingly, researchers have shown that the replacement of these metabolites in mice had a positive effect on reducing DSS-induced colitis and cytokine levels in the blood and brain. In addition, the replacement of these metabolites also significantly improved anxiety and depression-like behavior (110). This series of findings clearly demonstrates the important role of microbial metabolites in the “gut-immune-brain” communication of “comorbidities of mind and body”.

Furthermore, the gut microbiota is involved in neurotransmitter metabolism. At the subordinate level, Lactobacillus, Bifidobacterium, Escherichia, Enterococcus, and other species can produce neurotransmitters, neuropeptides, or other substances (such as metabolites) that can affect neural activity (125, 126). Lactobacillus and Bifidobacterium have been shown to produce GABA and Ach (127), while *L. rhamnosus* can regulate the central expression of GABA receptors in key regions of the mouse brain (128). The genus Bacteroides is associated not only with the occurrence of colitis but also with the expansion of its species, which mediates depressive-like behavior and impaired hippocampal neurogenesis by regulating tryptophan and neurotransmitter metabolism (129).

In summary, targeting the gut microbiota and its metabolites has great potential in developing new interventions to treat the intertwined pathology of the gut and brain. Identifying and characterizing microbial metabolites that affect the gut-brain axis can reveal new therapeutic targets. This leads to a promising path for future research and clinical application in the field of psychological gastrointestinal diseases. Meanwhile, more longitudinal and interventional clinical studies should be conducted to validate the findings of animal models.

## 4 New treatment strategies for IBD based on the brain-gut-microbiota axis

**Figure 2:**
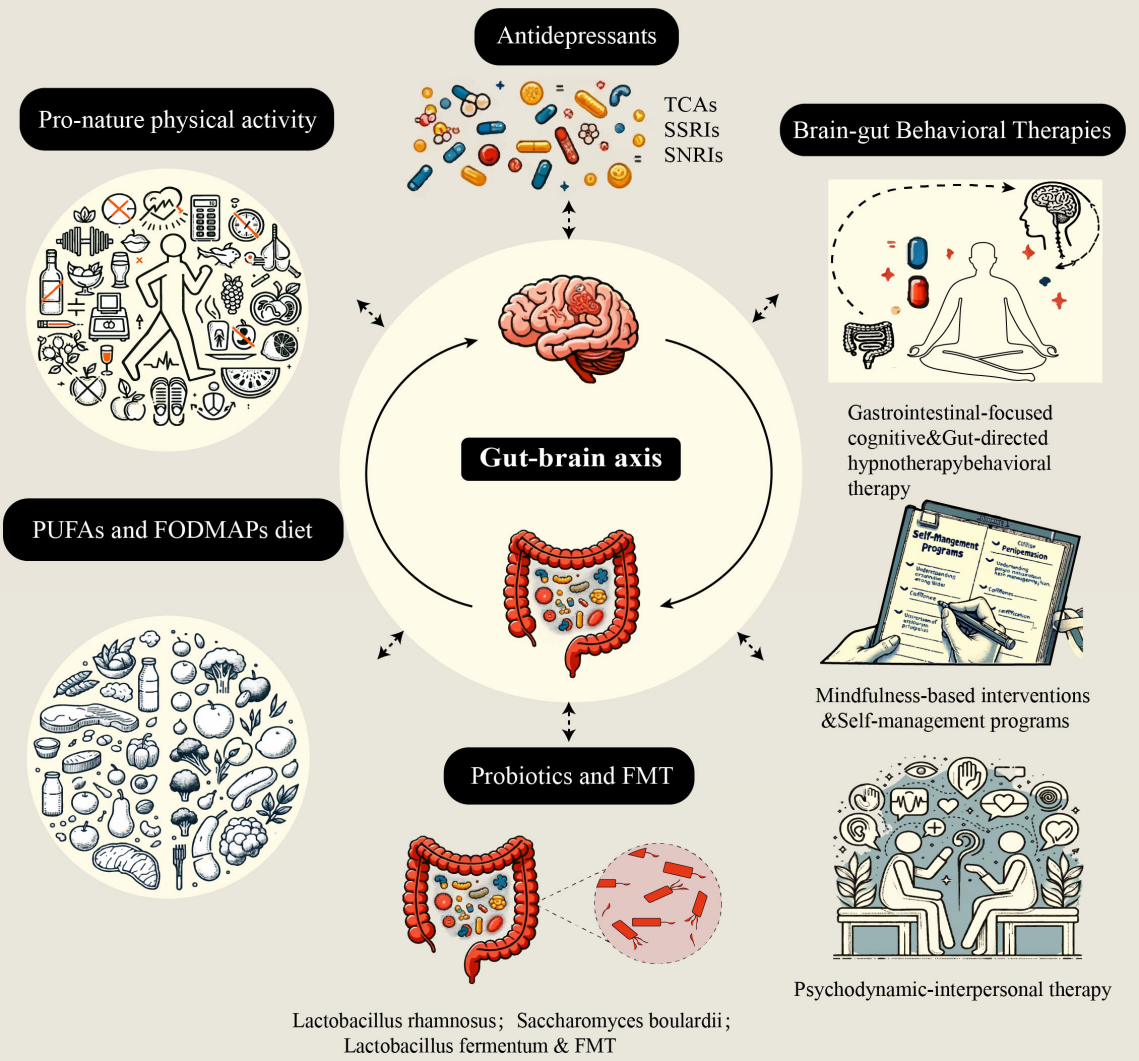
*Five nontraditional treatment strategies based on the brain-gut-microbiota axis.* These five treatment strategies aim to alleviate intestinal inflammation and improve psychological disorders by affecting different aspects of the brain-gut-microbiota axis. TCAs, tricyclic antidepressants; SSRIs, selective serotonin reuptake inhibitors; SNRIs, serotonin and norepinephrine reuptake inhibitors; FMT, fecal microbiota transplantation; PUFAs, polyunsaturated fatty acids; FODMAPs, fermentable oligosaccharides, disaccharides, monosaccharides, and polyols.

The possible mechanisms underlying the interaction between IBD and psychological disorders are described above. Therapeutic strategies targeting the gut microbiota, psychological disorders, neuroinflammation, and the immune microenvironment are therefore logical. Research has shown that the use of antidepressants, modulators of the gut microbiota, and other psychological interventions to treat “comorbidities” is often effective (**Figure 2**).

### 4.1 Antidepressants in IBD

To date, no antidepressant has been explicitly approved for the treatment of gastrointestinal diseases, but antidepressants are widely used as a main treatment method for disorders of gut–brain interaction (DGBIs), as shown in **Table 2**. Commonly used antidepressants include tricyclic antidepressants (TCAs), selective serotonin reuptake inhibitors (SSRIs), serotonin and norepinephrine reuptake inhibitors (SNRIs), and other antidepressants. Antidepressants can relieve gastrointestinal symptoms by regulating neurotransmitters such as serotonin, norepinephrine, and CRF, which play a role in intestinal motility and sensation (139). At the same time, by blocking the receptors 5-HT, norepinephrine, and several other neurotransmitters, antidepressants can also exert analgesic effects (140).

**Table 2:** The mechanism of action, benefits and side effects of antidepressants in IBD.

Drug type	Mechanism	Representative drugs	Impact on BGMA	Benefits for IBD populations	Side effects
TCAs (130)	Inhibit serotonin and norepinephrine reuptake	Promethazine, Clomipramine, Amitriptyline, Doxepin, Maprotiline	Reduce visceral sensitivity through anticholinergic effects to alleviate symptoms of abdominal pain	Improve abdominal pain, reduce visceral sensitivity, alleviate depression, and slow down gastrointestinal transmission	Dry mouth, dilated pupils, blurred vision, constipation, difficulty urinating, urinary retention, high intraocular pressure and tachycardia
SSNIs (131–133)	Inhibit serotonin reuptake	Fluoxetine, Paroxetine, Sertraline, Fluvoxamine, Citalopram, Escitalopram	Reduce the activation of microglia and the production of cytokines. Inhibit the activation and proliferation of antigen presenting cells to regulate adaptive immunity	Relieve anxiety and depression, improve psychological depression and insomnia	Nausea, vomiting, diarrhea and dizziness
SNRIs (134, 135)	Inhibit serotonin and norepinephrine reuptake	Venlafaxine, Duloxetine	Inhibition of descending nociceptive activity dependent on noradrenergic signaling in the brainstem to alleviate pain	Improve the symptoms of physical pain associated with depression and anxiety	Constipation, dry mouth, sedation, urinary retention and blurred vision
NaSSAs (136)	α2, 5-HT_2_ and 5-HT_3_ receptor antagonist	Mirtazapine	N/A	Improve symptoms of depression and insomnia, alleviate abdominal pain and diarrhea	Excessive sedation, dry mouth, weight gain, increased appetite, dizziness, fatigue
NDRIs (137)	Inhibit norepinephrine and dopamine reuptake	Bupropion	N/A	Preventive treatment of seasonal psychological disorders and antidepressant effects	Headache, insomnia, nausea, dry mouth, excessive sweating, anxiety, agitation and weight loss
Others (138)	Activate the 5-HT_1A_ receptor	Buspirone	Suppress neuroinflammation and regulate gut microbiota	Improve anxiety symptoms	Nausea, dizziness, tinnitus, headache and nervousness

In addition, antidepressants can reduce afferent signals from the gut and downregulate afferent visceral signals by affecting the function of the anterior cingulate cortex. Another theory suggests that long-term use of antidepressants can restore lost neurons and reduce the risk of depression recurrence (141, 142). A study revealed that after treatment with antidepressants, the level of BDNF gradually increased, and this increase seemed to be correlated with the degree of recovery from depression (141). To date, there are few data or low-level evidence obtained from the use of antidepressants for IBD. A prospective study involving 331 IBD patients revealed that after receiving antidepressant treatment (143), the number of patients (especially those with abnormal anxiety or depression scores at the time of study enrollment) receiving drug escalation therapy decreased. Similarly, in a retrospective case evaluation involving 58 IBD patients, compared with patients in the control group, patients taking various antidepressants had fewer relapses and corticosteroid courses within one year after starting antidepressant treatment (144).

### 4.2 Probiotics and FMT in IBD

Modulating the gut microbiota by administering probiotics is a potential strategy for the prevention and treatment of IBD. Several animal studies have shown that the use of probiotic strains can alleviate DSS-induced colitis, reduce systemic and CNS cytokine levels, restore inflammatory-related microflora dysregulation-related miRNA expression, and improve depressive and anxiety-like behavior (145, 146). Notably, *Faecalibacterium Prausnitzii*, a highly IBD-related species (with significantly decreased abundance in active IBD) (147, 148), could prevent anxiety and depression-like behavior in stressed rats (149). Research has found that after administration of *L. rhamnosus*, the expression of GABA Aα2 mRNA in the prefrontal cortex and amygdala decreased (150), thereby reducing plasma corticosterone levels. Moreover, supplementation with Lactobacillus strains can reduce stress-induced HPA activation and corticosterone, indirectly increasing the levels of BDNF in the hippocampus and 5-HT in the prefrontal cortex and frontal cortex of mice, which plays a role in preventing anxiety and depression (128).

In patient-based studies, probiotic treatment has been shown to significantly relieve IBD symptoms (**Table 3**). In CD patients, the administration of *Saccharomyces boulardii* is helpful for alleviating symptoms and reducing bowel sealing (151). In UC, Bifidobacterium and *Lactobacillus acidophilus* can maintain symptoms during low-activity periods (152). Moreover, the administration of *Lactobacillus fermentum* to UC patients can reduce the levels of NF-κB, IL-6, and TNF-α (153). Inconsistencies in the results of supplementation with the same probiotic strain can be triggered by the influence of flora activity and the presence of other strains in the host, indicating that compound probiotics often have greater therapeutic benefits for IBD than single strains. However, the results showing that probiotics directly reduce symptoms of anxiety and depression in IBD patients are mixed, showing contradictory results. A meta-analysis of randomized controlled trials (RCTs) in recent years has shown that the evidence obtained is insufficient to support the effectiveness of probiotics in treating psychological disorders in IBD patients, and including depressed and nondepressed individuals in the same analysis, as well as using different strains and treatment regimens, is still insufficient to demonstrate the direct effectiveness of probiotics in alleviating psychological disorders (161, 162). Other studies have reported the positive effects of probiotics on psychological, cognitive and emotional function; anxiety; and stress (163).

**Table 3:** Clinical trials of probiotics and FMT in the treatment of IBD.

Study	Country	Intervention (I)	Comparator (C)	Design	Follow-up	N (I/C)	Subject	Clinical response
Garcia et al 2008 (151)	Brazil	*S. boulardii* + baseline therapy	Placebo + baseline therapy	RCT	3 months	14/17	CD in remission period	*P*=0.0006 (*S. boulardii*); *P*= 0.12 (placebo)^1^
Kato et al 2004 (152)	Japan	BFM + baseline therapy	Placebo + baseline therapy	RCT	12 weeks	10/9	Active UC	*P* < 0.05^2^
David et al 2013 (153)	Ireland	*B. infantis* + baseline therapy	Placebo + baseline therapy	RCT	8 weeks	13/9	Active UC	*P*=0.0327 (CRP); 8/13 (*B. infantis*), 0/9 (Placebo)^3^
Hiroyuki et al 2016 (154)	Japan	BB536 + baseline therapy	Placebo + baseline therapy	RCT	8 weeks	24/23	Active UC	*P* < 0.05^4^
Shen et al 2024 (155)	China	BLE + baseline therapy	Placebo + baseline therapy	RCT	4 weeks	48/48	Active CD	47/48 (BLE), 41/48 (Placebo), *P*=0.027; *P* < 0.05 (L/M ratio)^5^
Samuel et al 2019 (156)	Australia	dFMT	aFMT	RCT	12 months	38/35	Active UC	12/38 (dFMT), 3/35 (aFMT), *P* = 0.03^6^
Sudarshan et al 2017 (157)	Australia	FMT	Placebo	RCT	8 weeks	41/40	Active UC	11/41 (FMT), 3/40 (Placebo), *P*=0.021^7^
Paul et al 2015 (158)	Canada	FMT + baseline therapy	Placebo + baseline therapy	RCT	6 weeks	38/37	Active UC	9/38 (FMT), 2/37 (Placebo), *P* <0.03^8^
Noortje et al 2015 (159)	Netherlands	dFMT	aFMT	RCT	12 weeks	17/20	Active UC	7/17 (dFMT), 5/20 (aFMT), *P*=0.29^9^
Sood et al 2019 (160)	India	FMT + baseline therapy	Placebo + baseline therapy	RCT	48 weeks	31/30	UC in clinical remission	*P*=0.026 (endoscopic remission); *P*=0.033 (histological remission)^10^

^1^Difference in the mean lactulose/mannitol ratio (used to evaluate intestinal permeability) between the end of the third month and the beginning of treatment. S. boulardii added to baseline therapy improved intestinal permeability in these patients. S. boulardii: Saccharomyces boulardii; RCT: Randomized controlled trial; CD: Crohn’s disease.

^2^The average CAI score in the BFM group had significantly decreased to 3.7 ± 0.4 by 12 weeks (P < 0.001). In the placebo group, the CAI score had decreased to 5.8 ± 0.8 at 12 weeks (P < 0.05). The CAI score in the BFM group was significantly lower than that of the placebo group at 12 weeks (P < 0.05). BFM: biﬁdobacteria-fermented milk; CAI: clinical activity index; UC: ulcerative colitis.

^3^B. infantis 35624-feeding induces a reduction in absolute CRP levels in UC patients compared with placebo-fed patients; Patients with UC achieve reductions in plasma CRP, TNF-α and IL-6 levels following feeding with B. infantis 35624 compared with placebo-fed patients. B. infantis: Bifidobacteria infantis; CRP: C-reactive protein; TNF-α: tumor necrosis factor; IL, interleukin.

^4^BB536: Rectal bleeding (0.79 ± 0.19 at baseline vs 0.5 ± 0.17 at week 8; P=0.038); Mucosal findings (2.23 ± 0.51 at baseline vs 1.73 ± 0.6 at week 8; P=0.017); Endoscopic index scores (6.7 ± 0.5 at baseline vs 3.7 ± 0.6 at week 8; P<0.01); Mayo subscores (2.2 ± 0.10 at baseline vs 1.5 ± 0.73 at week 8; P<0.01), whereas there was no significant decrease in the placebo group. BB536: Bifidobacterium longum 536.

^5^The total clinical effective rate of patients in the observation group (97.91%) was elevated in comparison with that of the control group (85.42%) (P=0.027). After the treatment, the levels of urinary lactulose/mannitol ratio in the two groups were decreased versus those prior to the treatment, and these factors in the observation group were reduced versus those in the control group (P < 0.05).

^6^The primary end point of steroid-free remission was achieved in more participants who received dFMT compared with aFMT (12/38 [32%] vs 3/35 [9%]; difference, 23% [95% CI, 4%-42%]; odds ratio [OR], 5.0 [95% CI, 1.2-20.1]; P = 0.03). dFMT: donor fecal microbiota transplantation; aFMT: autologous fecal microbiota transplantation.

^7^The primary outcome (steroid-free clinical remission with endoscopic remission or response) was achieved in 11 (27%) of 41 patients allocated FMT versus three (8%) of 40 who were assigned placebo (risk ratio 3·6, 95% CI 1·1–11·9; P=0.021).

^8^There was a statistically significant effect of FMT on inducing remission in UC, with 9 of 38 (24%) patients in the FMT arm vs 2 of 37 (5%) in the placebo arm in remission at the end of treatment (P<0.03).

^9^In the per-protocol analysis, 7 of 17 patients who received FMT from healthy donors (41.2%) and 5 of 20 controls (25.0%) achieved the primary end point (P=0 .29). There was no statistically significant difference in clinical and endoscopic remission between patients with UC who received fecal transplants from healthy donors and those who received their own fecal microbiota.

^10^Endpoints of endoscopic remission (FMT: 18/31 [58.1%] versus placebo: 8/30 [26.7%], P= 0.026) and histological remission (FMT: 14/31 [45.2%] versus placebo: 5/30 [16.7%], P= 0. 033) were achieved in a significantly higher number of patients with FMT.

FMT is a new microbiome-based therapy in which feces are transferred from healthy donors to the intestinal tract of patients through endoscopy or colonoscopy to reduce intestinal dysbiosis (164). Compared with prebiotics, probiotics, and antibiotics, FMT has a more direct and efficient effect on restoring intestinal microecological balance. According to some literature reports, with the deepening of researchers’ understanding of the MGBA, FMT has good clinical efficacy and promising prospects in treating neuropsychiatric diseases, especially refractory psychiatric diseases (164). Some studies have shown that compared with the use of a placebo, the use of FMT for the treatment of IBD significantly alleviates endoscopic and clinical symptoms (165). At the same time, the therapeutic potential of FMT also manifests in the alleviation of some extraintestinal symptoms, such as anxiety, depression, and autism (166). Notably, negative emotions can also occur with FMT; for example, anxiety-like behaviors caused by chronic stress can appear in recipient mice after FMT with stressed mice as donors (69). Similarly, mice receiving a fecal transplant from patients with irritable bowel syndrome and anxiety not only exhibit intestinal symptoms but also exhibit more pronounced anxiety-like behaviors (167). Although the treatment of FMT is very individualized and its efficacy is very rapid compared with that of oral probiotics, the long-term effects and potential risks of FMT are still unclear, and large-scale follow-ups in the population are still needed to clarify the long-term prognosis.

### 4.3 Brain-gut behavioral therapies

A prospective longitudinal population-based study revealed that psychological factors contributed more to the perception of health in the IBD cohort than in the control group (168). However, inactive IBD patients were very similar to non-IBD control group patients. Based on the above results, it suggests that psychological disorders are not specifically associated with the disease itself but mainly affect disease activity. Among the IBD patient samples observed in a gastroenterology department, it was found that the severity of the disease and psychological disorders independently led to impaired QOL (169). In another study, after appropriate psychological assistance, the intestinal and systemic symptoms of IBD patients were alleviated, the level of activity participation increased, symptom tolerance increased, pain decreased, perceived stress decreased, and the number of visits to the gastroenterology department decreased (170). Therefore, in addition to considering drug treatment, how to improve the psychological health and cognitive function of IBD patients through wider daily communication on their diagnosis, treatment and QOL, especially by establishing a positive, trusting and harmonious doctor−patient relationship, is worth considering for researchers and medical staff.

Currently, the most widely studied psychological gastroenterological intervention is brain-gut behavioral therapy (BGBT) (171), which includes gastrointestinal-focused cognitive−behavioral therapy, gut-directed hypnotherapy, mindfulness-based interventions, self-management programs, and psychodynamic-interpersonal therapy. Based on the idea that “thoughts, emotions, and behaviors can be learned and influence both the symptoms experienced by patients and their perception of these symptoms”, gastrointestinal-focused cognitive−behavioral therapy aims to achieve self-stress management by adjusting cognitive processes, practicing mindfulness, and receiving stress reduction training (172). Gut-directed hypnotherapy is a method used by professional clinicians to induce a highly focused state of consciousness in DGBI patients through video calls and digital means to increase their acceptance of posthypnosis suggestions (173). This can improve the poor cognition and extraintestinal manifestations of DGBI patients to a certain extent (174). Self-management programs aim to help patients realize that they are the managers of their own disease and need to actively participate in the management of their disease process (175). By establishing a workbook, improving their understanding of disease-inducing factors, correcting erroneous beliefs, and boosting self-confidence and reducing stress, patients can achieve their goals. Psychodynamic-interpersonal therapy is the most important of the five methods (176). Research has shown that a strong, trusting, and cooperative relationship between doctors and patients is crucial for patients to reduce their negative emotions caused by physical symptoms, as poor interpersonal relationships have become a stumbling block in their lives. Through effective communication, full empathy, active listening, and recognition of patients’ experiences and concerns, patients can discover their own value and increase their enthusiasm for participation (177).

Through the above five methods, patients can better understand and manage their emotions and alleviate the emotional stress and anxiety caused by the disease. At the same time, patients can learn to cope with the decline in QOL related to IBD, including dealing with social isolation, physical discomfort, dietary restrictions, and medical procedures. Poor disease control may affect QOL, while BGBT can help increase patients’ life satisfaction. In addition, lifestyle advice on diet, exercise, and sleep is also provided to patients through BGBT. When experiencing pain and discomfort, the clever use of relaxation techniques, meditation, and deep breathing can be beneficial for alleviating physical and mental stress. Finally, a good and trusting doctor−patient relationship can help increase patient compliance and their understanding of their treatment options, thereby increasing their likelihood of following medical advice. Therefore, it is necessary to use both drug therapy and BGBT for comprehensive treatment.

### 4.4 PUFAs and FODMAPs diet

The development of a reasonable dietary strategy for IBD patients requires long-term adherence, which is often overlooked by patients and clinicians. Epidemiological surveys have shown that diets containing a large amount of animal fat and a small amount of fruits and vegetables may be associated with an increased risk of IBD (178). In mouse models, high-fat diets, especially those rich in saturated fatty acids, also increase inflammation, while supplementation with omega-3 long-chain fatty acids can significantly reduce the production of cyclooxygenase-2 (COX-2), IL-6, iNOS, and TNF-α in the colon, thereby reducing intestinal inflammation (179). It has been reported that consuming diets rich in omega-3 and omega-6 polyunsaturated fatty acids (PUFAs) in the early stages of external stress can regulate the composition of the gut microbiota and the abundance of gut microorganisms, for example, by increasing probiotics such as bifidobacteria and lactobacilli (180). In addition, studies have confirmed that omega-3 fatty acid supplementation can reduce the excessive activation of the HPA axis in adolescent female rats under stressful conditions and improve their cognitive ability in adulthood (181, 182). These findings suggest that rationalizing dietary consumption has the benefit of reducing stress and depressive behavior.

A diet low in fermentable oligosaccharides, disaccharides, monosaccharides, and polyols (FODMAPs) is recommended as a first-line treatment for IBS (183). The occurrence of IBD is associated with food allergies or overconsumption of specific types of carbohydrates. By reducing the intake of FODMAPs, it is possible to reduce fermentation and gas production in the gut, thereby alleviating symptoms such as bloating, abdominal pain, and diarrhea (184). In a randomized, placebo-controlled trial, researchers found that compared to a sham dietary advice control group, patients with inactive IBD who received advice on a low FODMAP diet experienced improvements in their intestinal symptoms and QOL (185). This finding may be related to the fact that a low FODMAP diet changes the diversity of the gut microbiota, especially the abundance of bifidobacteria, but has no significant effect on inflammatory markers (186).

### 4.5 Pro-nature physical activity

As a physical and mental disease, IBD often involves many psychological disorders, including fatigue, depression, anxiety, and decreased QOL, in addition to the intestinal symptoms of the disease itself (187, 188). In addition to controlling inflammation, adhering to a healthy lifestyle (moderate-to-high intensity physical activity, weight control, limited alcohol consumption, no smoking, and Mediterranean diet) is associated with lower all-cause mortality in elderly IBD patients. In a recent review by the Crohn’s and Colitis Organization of Europe, it was confirmed that patients with IBD, especially CD, who suffer from “comorbid physical and mental illnesses”, can benefit from physical activity. A lack of physical activity often leads to overweight and even obesity (189–191). During long-term follow-up, obese IBD patients were found to have a lower clinical remission rate of drug treatment and higher anxiety, depression, fatigue, and pain scores, as measured by the PROMIS (192). At the same time, the recurrence rate of the disease, the rate of repeat visits to the gastroenterology clinic, and the incidence of complications were significantly greater in these patients than in those who participated in sufficient physical activity (193).

Lou found that combining structured physical activity with a lifestyle that fully exposes people to nature, or “pro-nature physical activity”, can effectively improve the “physical and mental comorbidities” of patients with DGBIs (194). This “green prescription” not only embodies the medical treatment concept from a holistic viewpoint, but also provides a new perspective and means for treating diseases related to stress and psychological disorders (195). Although there are few studies on the mechanism by which physical activity alleviates the “physical and mental comorbidities” of IBD patients, and most of them have insufficient sample sizes, existing research shows that physical activity can regulate the diversity of gut microorganisms and intervene in specific gut microbiota and bacterial metabolites (such as SCFAs) to affect the disease (196–198). Physical activity may lead to epigenetic modifications and regulate the transcription of key genes responsible for acute and chronic benefits from physical activity. Moreover, physical activity can reduce visceral fat content and the expression of proinflammatory factors (such as TNF-α and IL-6) and increase intestinal mucosal immunity, epithelial integrity, and tissue regeneration. Among them, pro-nature physical activity can regulate the MGBA; increase the abundance of Firmicutes and Akkermansia bacteria; increase SCFAs levels; and further affect the HPA axis, BDNF, and serotonin pathways involved in bidirectional gut-brain communication, thereby maintaining body homeostasis and reducing psychological stress (194).

Although Lou’s research confirms that nature-based physical activity is a more efficient form of exercise, it does not compare specific types of exercise (such as basketball, running, and swimming), and there is no universal standard for exercise intensity and frequency. Therefore, in future observational studies, additional evaluation indicators need to be established to determine the optimal exercise regimen. Despite this, the proposal of nature-based physical activity still provides direction for patients, clinicians, health departments, and urban construction departments. Nature-based physical activity not only is beneficial to humans but also has great significance for the harmonious coexistence of man and nature, as well as the restoration and protection of the natural environment.

## 5 Conclusion

In this review, we have explored the possible mechanisms and influencing factors of the activation of the gut-brain pathway leading to psychological changes in IBD patients, and we have summarized the bidirectional regulatory role of the BGMA. Additionally, focusing on the relationship between psychological changes before and after the onset of IBD and the development of the disease, we have proposed the concept of “psychosomatic treatment”. For readers who are interested in psychological stress and the development of diseases, we advocate interdisciplinary interaction, especially the intersection of psychological stress, neuroendocrinology, neuroimmunology, and traditional pathophysiology. We have also analyzed current laboratory and clinical data on this topic, as well as potential treatment strategies and disease progression management strategies that may be useful for these patients. In summary, the mechanisms by which disruption of the gut-brain pathway leads to psychological disorders in IBD patients are diverse, with the gut microbiota, nerves, inflammation, and immune microenvironment all playing important roles. Although there are many animal models supporting the above research findings, studies in human patients are still relatively limited; therefore, more clinical research is needed to verify the applicability of these findings to humans. We need to better understand the interactions between the brain and the gut, including the intersection of neural, immune, and metabolic pathways, which will help us better identify potential therapeutic targets. In addition, in terms of clinical application, we need to develop more targeted treatment strategies to meet the individualized needs of patients with IBD and psychological disorders and achieve the goal of “psychosomatic treatment”.

## Author contributions

SW: Writing – original draft. SZ: Writing – original draft. ZH: Writing – original draft, Writing – review & editing. BY: Writing – original draft. YX: Writing – original draft. YuL: Writing – original draft. YC: Writing – original draft. ZJ: Writing – original draft. YaL: Writing – original draft. QC: Writing – original draft. YX: Writing – original draft. QZ: Writing – review & editing. YW: Writing – review & editing.

## References

[1] Baumgart DC Carding SR. Inflammatory bowel disease: cause and immunobiology. Lancet (London England). (2007) 369:1627–40. doi: 10.1016/s0140-6736(07)60750-8 17499605

[2] Levine A Sigall Boneh R Wine E. Evolving role of diet in the pathogenesis and treatment of inflammatory bowel diseases. Gut. (2018) 67:1726–38. doi: 10.1136/gutjnl-2017-315866 29777041

[3] Kaplan GG. The global burden of ibd: from 2015 to 2025. Nat Rev Gastroenterol Hepatol. (2015) 12:720–7. doi: 10.1038/nrgastro.2015.150 26323879

[4] Kaplan GG Ng SC. Globalisation of inflammatory bowel disease: perspectives from the evolution of inflammatory bowel disease in the uk and China. Lancet Gastroenterol Hepatol. (2016) 1:307–16. doi: 10.1016/s2468-1253(16)30077-2 28404201

[5] Zois CD Katsanos KH Kosmidou M Tsianos EV. Neurologic manifestations in inflammatory bowel diseases: current knowledge and novel insights. J Crohn's colitis. (2010) 4:115–24. doi: 10.1016/j.crohns.2009.10.005 21122494

[6] Levine JS Burakoff R. Extraintestinal manifestations of inflammatory bowel disease. Gastroenterol Hepatol. (2011) 7:235–41. PMC3127025 21857821

[7] Gracie DJ Guthrie EA Hamlin PJ Ford AC. Bi-directionality of brain-gut interactions in patients with inflammatory bowel disease. Gastroenterology. (2018) 154:1635–46.e3. doi: 10.1053/j.gastro.2018.01.027 29366841

[8] Blackwell J Saxena S Petersen I Hotopf M Creese H Bottle A. Depression in individuals who subsequently develop inflammatory bowel disease: A population-based nested case-control study. Gut. (2021) 70:1642–8. doi: 10.1136/gutjnl-2020-322308 33109601

[9] Neuendorf R Harding A Stello N Hanes D Wahbeh H. Depression and anxiety in patients with inflammatory bowel disease: A systematic review. J Psychosom Res. (2016) 87:70–80. doi: 10.1016/j.jpsychores.2016.06.001 27411754

[10] Barberio B Zamani M Black CJ Savarino EV Ford AC. Prevalence of symptoms of anxiety and depression in patients with inflammatory bowel disease: A systematic review and meta-analysis. Lancet Gastroenterol Hepatol. (2021) 6:359–70. doi: 10.1016/s2468-1253(21)00014-5 33721557

[11] Frolkis AD Vallerand IA Shaheen AA Lowerison MW Swain MG Barnabe C. Depression increases the risk of inflammatory bowel disease, which may be mitigated by the use of antidepressants in the treatment of depression. Gut. (2019) 68:1606–12. doi: 10.1136/gutjnl-2018-317182 30337374

[12] Ananthakrishnan AN Khalili H Pan A Higuchi LM de Silva P Richter JM. Association between depressive symptoms and incidence of crohn's disease and ulcerative colitis: results from the nurses' Health study. Clin Gastroenterol Hepatol. (2013) 11:57–62. doi: 10.1016/j.cgh.2012.08.032 22944733 PMC3587728

[13] Mikocka-Walus A Knowles SR Keefer L Graff L. Controversies revisited: A systematic review of the comorbidity of depression and anxiety with inflammatory bowel diseases. Inflammatory bowel Dis. (2016) 22:752–62. doi: 10.1097/mib.0000000000000620 26841224

[14] Kurina LM Goldacre MJ Yeates D Gill LE. Depression and anxiety in people with inflammatory bowel disease. J Epidemiol Community Health. (2001) 55:716–20. doi: 10.1136/jech.55.10.716 PMC1731788 11553654

[15] Fairbrass KM Lovatt J Barberio B Yuan Y Gracie DJ Ford AC. Bidirectional brain-gut axis effects influence mood and prognosis in ibd: A systematic review and meta-analysis. Gut. (2022) 71:1773–80. doi: 10.1136/gutjnl-2021-325985 34725197

[16] Butler MI Cryan JF Dinan TG. Man and the microbiome: A new theory of everything? Annu Rev Clin Psychol. (2019) 15:371–98. doi: 10.1146/annurev-clinpsy-050718-095432 30786244

[17] Collins SM Bercik P. The relationship between intestinal microbiota and the central nervous system in normal gastrointestinal function and disease. Gastroenterology. (2009) 136:2003–14. doi: 10.1053/j.gastro.2009.01.075 19457424

[18] Bernstein CN. The brain-gut axis and stress in inflammatory bowel disease. Gastroenterol Clinics North America. (2017) 46:839–46. doi: 10.1016/j.gtc.2017.08.006 29173525

[19] Kinugasa H Hiraoka S Oka S Okada H. Ulcerative colitis associated with a mixed neuroendocrine-non-neuroendocrine neoplasm. Internal Med (Tokyo Japan). (2020) 59:2085–6. doi: 10.2169/internalmedicine.4609-20 PMC7492109 32448840

[20] Kiank C Taché Y Larauche M. Stress-related modulation of inflammation in experimental models of bowel disease and post-infectious irritable bowel syndrome: role of corticotropin-releasing factor receptors. Brain behavior Immun. (2010) 24:41–8. doi: 10.1016/j.bbi.2009.08.006 PMC2962412 19698778

[21] Walker JR Ediger JP Graff LA Greenfeld JM Clara I Lix L. The manitoba ibd cohort study: A population-based study of the prevalence of lifetime and 12-month anxiety and mood disorders. Am J Gastroenterol. (2008) 103:1989–97. doi: 10.1111/j.1572-0241.2008.01980.x 18796096

[22] Bailey MT Engler H Sheridan JF. Stress induces the translocation of cutaneous and gastrointestinal microflora to secondary lymphoid organs of C57bl/6 mice. J neuroimmunology. (2006) 171:29–37. doi: 10.1016/j.jneuroim.2005.09.008 16253348

[23] Pariante CM Lightman SL. The hpa axis in major depression: classical theories and new developments. Trends Neurosci. (2008) 31:464–8. doi: 10.1016/j.tins.2008.06.006 18675469

[24] Wang SL Shao BZ Zhao SB Chang X Wang P Miao CY. Intestinal autophagy links psychosocial stress with gut microbiota to promote inflammatory bowel disease. Cell Death Dis. (2019) 10:391. doi: 10.1038/s41419-019-1634-x 31564717 PMC6766473

[25] Vicario M Guilarte M Alonso C Yang P Martínez C Ramos L. Chronological assessment of mast cell-mediated gut dysfunction and mucosal inflammation in a rat model of chronic psychosocial stress. Brain behavior Immun. (2010) 24:1166–75. doi: 10.1016/j.bbi.2010.06.002 20600818

[26] Overman EL Rivier JE Moeser AJ. Crf induces intestinal epithelial barrier injury *via* the release of mast cell proteases and tnf-A. PloS One. (2012) 7:e39935. doi: 10.1371/journal.pone.0039935 22768175 PMC3386952

[27] Konturek PC Brzozowski T Konturek SJ. Stress and the gut: pathophysiology, clinical consequences, diagnostic approach and treatment options. J Physiol Pharmacol. (2011) 62:591–9. 22314561

[28] Im E Rhee SH Park YS Fiocchi C Taché Y Pothoulakis C. Corticotropin-releasing hormone family of peptides regulates intestinal angiogenesis. Gastroenterology. (2010) 138:2457–67. doi: 10.1053/j.gastro.2010.02.055 PMC2883634 20206175

[29] Wood SK Woods JH. Corticotropin-releasing factor receptor-1: A therapeutic target for cardiac autonomic disturbances. Expert Opin Ther Targets. (2007) 11:1401–13. doi: 10.1517/14728222.11.11.1401 18028006

[30] Goode T O'Connell J Anton P Wong H Reeve J O'Sullivan GC. Neurokinin-1 receptor expression in inflammatory bowel disease: molecular quantitation and localisation. Gut. (2000) 47:387–96. doi: 10.1136/gut.47.3.387 PMC1728039 10940277

[31] Iftikhar K Siddiq A Baig SG Zehra S. Substance P: A neuropeptide involved in the psychopathology of anxiety disorders. Neuropeptides. (2020) 79:101993. doi: 10.1016/j.npep.2019.101993 31735376

[32] Stucchi AF Shofer S Leeman S Materne O Beer E McClung J. Nk-1 antagonist reduces colonic inflammation and oxidative stress in dextran sulfate-induced colitis in rats. Am J Physiol Gastrointestinal liver Physiol. (2000) 279:G1298–306. doi: 10.1152/ajpgi.2000.279.6.G1298 11093954

[33] Simeonidis S Castagliuolo I Pan A Liu J Wang CC Mykoniatis A. Regulation of the nk-1 receptor gene expression in human macrophage cells via an nf-kappa B site on its promoter. Proc Natl Acad Sci United States America. (2003) 100:2957–62. doi: 10.1073/pnas.0530112100 PMC151448 12594338

[34] Sideri A Bakirtzi K Shih DQ Koon HW Fleshner P Arsenescu R. Substance P mediates pro-inflammatory cytokine release form mesenteric adipocytes in inflammatory bowel disease patients. Cell Mol Gastroenterol Hepatol. (2015) 1:420–32. doi: 10.1016/j.jcmgh.2015.03.003 PMC4629258 26543894

[35] Mawe GM Hoffman JM. Serotonin signalling in the gut–functions, dysfunctions and therapeutic targets. Nat Rev Gastroenterol Hepatol. (2013) 10:473–86. doi: 10.1038/nrgastro.2013.105 PMC4048923 23797870

[36] Martel F Monteiro R Lemos C. Uptake of Serotonin at the Apical and Basolateral Membranes of Human Intestinal Epithelial (Caco-2) Cells Occurs through the Neuronal Serotonin Transporter (Sert). J Pharmacol Exp Ther. (2003) 306:355–62. doi: 10.1124/jpet.103.049668 12682218

[37] Linden DR Chen JX Gershon MD Sharkey KA Mawe GM. Serotonin availability is increased in mucosa of Guinea pigs with tnbs-induced colitis. Am J Physiol Gastrointestinal liver Physiol. (2003) 285:G207–16. doi: 10.1152/ajpgi.00488.2002 12646422

[38] Bertrand PP. Real-time detection of serotonin release from enterochromaffin cells of the Guinea-pig ileum. Neurogastroenterol Motil. (2004) 16:511–4. doi: 10.1111/j.1365-2982.2004.00572.x 15500507

[39] Wan M Ding L Wang D Han J Gao P. Serotonin: A potent immune cell modulator in autoimmune diseases. Front Immunol. (2020) 11:186. doi: 10.3389/fimmu.2020.00186 32117308 PMC7026253

[40] Brumovsky P Shi TS Landry M Villar MJ Hökfelt T. Neuropeptide tyrosine and pain. Trends Pharmacol Sci. (2007) 28:93–102. doi: 10.1016/j.tips.2006.12.003 17222466

[41] El-Salhy M Grimelius L Wilander E Ryberg B Terenius L Lundberg JM. Immunocytochemical identification of polypeptide yy (Pyy) cells in the human gastrointestinal tract. Histochemistry. (1983) 77:15–23. doi: 10.1007/bf00496632 6341321

[42] Shibata M Hisajima T Nakano M Goris RC Funakoshi K. Morphological relationships between peptidergic nerve fibers and immunoglobulin a-producing lymphocytes in the mouse intestine. Brain behavior Immun. (2008) 22:158–66. doi: 10.1016/j.bbi.2007.08.013 17931829

[43] Shimizu H Bray GA. Effects of neuropeptide Y on norepinephrine and serotonin metabolism in rat hypothalamus in vivo. Brain Res Bull. (1989) 22:945–50. doi: 10.1016/0361-9230(89)90004-x 2477116

[44] ten Bokum AM Hofland LJ van Hagen PM. Somatostatin and somatostatin receptors in the immune system: A review. Eur Cytokine network. (2000) 11:161–76. 10903795

[45] Kaszaki J Erces D Varga G Szabó A Vécsei L Boros M. Kynurenines and intestinal neurotransmission: the role of N-methyl-D-aspartate receptors. J Neural Transm (Vienna Austria 1996). (2012) 119:211–23. doi: 10.1007/s00702-011-0658-x 21617892

[46] Krieglstein CF Cerwinka WH Laroux FS Salter JW Russell JM Schuermann G. Regulation of murine intestinal inflammation by reactive metabolites of oxygen and nitrogen: divergent roles of superoxide and nitric oxide. J Exp Med. (2001) 194:1207–18. doi: 10.1084/jem.194.9.1207 PMC2195977 11696587

[47] Viviani B Boraso M Marchetti N Marinovich M. Perspectives on neuroinflammation and excitotoxicity: A neurotoxic conspiracy? Neurotoxicology. (2014) 43:10–20. doi: 10.1016/j.neuro.2014.03.004 24662010

[48] Radesäter AC Johansson S Oberg C Luthman J. The vitamin-E analog trolox and the nmda antagonist mk-801 protect pyramidal neurons in hippocampal slice cultures from il-1beta-induced neurodegeneration. Neurotoxicity Res. (2003) 5:433–42. doi: 10.1007/bf03033173 14715447

[49] Niesler B Kuerten S Demir IE Schäfer KH. Disorders of the enteric nervous system - a holistic view. Nat Rev Gastroenterol Hepatol. (2021) 18:393–410. doi: 10.1038/s41575-020-00385-2 33514916

[50] Dothel G Barbaro MR Boudin H Vasina V Cremon C Gargano L. Nerve fiber outgrowth is increased in the intestinal mucosa of patients with irritable bowel syndrome. Gastroenterology. (2015) 148:1002–11.e4. doi: 10.1053/j.gastro.2015.01.042 25655556

[51] Lindgren S Lilja B Rosén I Sundkvist G. Disturbed autonomic nerve function in patients with crohn's disease. Scandinavian J Gastroenterol. (1991) 26:361–6. doi: 10.3109/00365529108996495 2034989

[52] Maule S Pierangeli G Cevoli S Grimaldi D Gionchetti P Barbara G. Sympathetic hyperactivity in patients with ulcerative colitis. Clin autonomic Res. (2007) 17:217–20. doi: 10.1007/s10286-007-0425-0 17574503

[53] Johnson JD Campisi J Sharkey CM Kennedy SL Nickerson M Greenwood BN. Catecholamines mediate stress-induced increases in peripheral and central inflammatory cytokines. Neuroscience. (2005) 135:1295–307. doi: 10.1016/j.neuroscience.2005.06.090 16165282

[54] Kyösola K Penttilä O Salaspuro M. Rectal mucosal adrenergic innervation and enterochromaffin cells in ulcerative colitis and irritable colon. Scandinavian J Gastroenterol. (1977) 12:363–7. doi: 10.3109/00365527709180942 867000

[55] Straub RH Stebner K Härle P Kees F Falk W Schölmerich J. Key role of the sympathetic microenvironment for the interplay of tumour necrosis factor and interleukin 6 in normal but not in inflamed mouse colon mucosa. Gut. (2005) 54:1098–106. doi: 10.1136/gut.2004.062877 PMC1774899 15845563

[56] Bonaz B Sinniger V Pellissier S. Anti-inflammatory properties of the vagus nerve: potential therapeutic implications of vagus nerve stimulation. J Physiol. (2016) 594:5781–90. doi: 10.1113/jp271539 PMC5063949 27059884

[57] Meregnani J Clarençon D Vivier M Peinnequin A Mouret C Sinniger V. Anti-inflammatory effect of vagus nerve stimulation in a rat model of inflammatory bowel disease. Autonomic Neurosci basic Clin. (2011) 160:82–9. doi: 10.1016/j.autneu.2010.10.007 21071287

[58] Koch C Wilhelm M Salzmann S Rief W Euteneuer F. A meta-analysis of heart rate variability in major depression. Psychol Med. (2019) 49:1948–57. doi: 10.1017/s0033291719001351 31239003

[59] Han W Tellez LA Perkins MH Perez IO Qu T Ferreira J. A neural circuit for gut-induced reward. Cell. (2018) 175:665–78.e23. doi: 10.1016/j.cell.2018.08.049 30245012 PMC6195474

[60] Taché Y Bonaz B. Corticotropin-releasing factor receptors and stress-related alterations of gut motor function. J Clin Invest. (2007) 117:33–40. doi: 10.1172/jci30085 17200704 PMC1716215

[61] Bonaz BL Bernstein CN. Brain-gut interactions in inflammatory bowel disease. Gastroenterology. (2013) 144:36–49. doi: 10.1053/j.gastro.2012.10.003 23063970

[62] Liu B Wanders A Wirdefeldt K Sjölander A Sachs MC Eberhardson M. Vagotomy and subsequent risk of inflammatory bowel disease: A nationwide register-based matched cohort study. Alimentary Pharmacol Ther. (2020) 51:1022–30. doi: 10.1111/apt.15715 32319125

[63] Martelli D Farmer DG Yao ST. The splanchnic anti-inflammatory pathway: could it be the efferent arm of the inflammatory reflex? Exp Physiol. (2016) 101:1245–52. doi: 10.1113/ep085559 27377300

[64] Rosas-Ballina M Olofsson PS Ochani M Valdés-Ferrer SI Levine YA Reardon C. Acetylcholine-synthesizing T cells relay neural signals in a vagus nerve circuit. Sci (New York NY). (2011) 334:98–101. doi: 10.1126/science.1209985 PMC4548937 21921156

[65] Peter J Fournier C Durdevic M Knoblich L Keip B Dejaco C. A microbial signature of psychological distress in irritable bowel syndrome. Psychosomatic Med. (2018) 80:698–709. doi: 10.1097/psy.0000000000000630 PMC6250280 30095672

[66] Goodhand JR Wahed M Mawdsley JE Farmer AD Aziz Q Rampton DS. Mood disorders in inflammatory bowel disease: relation to diagnosis, disease activity, perceived stress, and other factors. Inflammatory bowel Dis. (2012) 18:2301–9. doi: 10.1002/ibd.22916 22359369

[67] Cristofori F Dargenio VN Dargenio C Miniello VL Barone M Francavilla R. Anti-inflammatory and immunomodulatory effects of probiotics in gut inflammation: A door to the body. Front Immunol. (2021) 12:578386. doi: 10.3389/fimmu.2021.578386 33717063 PMC7953067

[68] Adamantidis A. How the gut talks to the brain. Sci (New York NY). (2022) 376:248–9. doi: 10.1126/science.abo7933 35420955

[69] Li N Wang Q Wang Y Sun A Lin Y Jin Y. Fecal microbiota transplantation from chronic unpredictable mild stress mice donors affects anxiety-like and depression-like behavior in recipient mice *via* the gut microbiota-inflammation-brain axis. Stress (Amsterdam Netherlands). (2019) 22:592–602. doi: 10.1080/10253890.2019.1617267 31124390

[70] Bailey MT Dowd SE Galley JD Hufnagle AR Allen RG Lyte M. Exposure to a social stressor alters the structure of the intestinal microbiota: implications for stressor-induced immunomodulation. Brain behavior Immun. (2011) 25:397–407. doi: 10.1016/j.bbi.2010.10.023 PMC3039072 21040780

[71] Bailey MT. The contributing role of the intestinal microbiota in stressor-induced increases in susceptibility to enteric infection and systemic immunomodulation. Hormones Behav. (2012) 62:286–94. doi: 10.1016/j.yhbeh.2012.02.006 22366706

[72] Bharwani A Mian MF Foster JA Surette MG Bienenstock J Forsythe P. Structural & Functional consequences of chronic psychosocial stress on the microbiome & Host. Psychoneuroendocrinology. (2016) 63:217–27. doi: 10.1016/j.psyneuen.2015.10.001 26479188

[73] Sun L Zhang H Cao Y Wang C Zhao C Wang H. Fluoxetine ameliorates dysbiosis in a depression model induced by chronic unpredicted mild stress in mice. Int J Med Sci. (2019) 16:1260–70. doi: 10.7150/ijms.37322 PMC6775263 31588192

[74] Tian T Xu B Qin Y Fan L Chen J Zheng P. Clostridium butyricum miyairi 588 has preventive effects on chronic social defeat stress-induced depressive-like behaviour and modulates microglial activation in mice. Biochem Biophys Res Commun. (2019) 516:430–6. doi: 10.1016/j.bbrc.2019.06.053 31227215

[75] Allen JM Mackos AR Jaggers RM Brewster PC Webb M Lin CH. Psychological stress disrupts intestinal epithelial cell function and mucosal integrity through microbe and host-directed processes. Gut Microbes. (2022) 14:2035661. doi: 10.1080/19490976.2022.2035661 35184677 PMC8865257

[76] Goto Y Panea C Nakato G Cebula A Lee C Diez MG. Segmented filamentous bacteria antigens presented by intestinal dendritic cells drive mucosal th17 cell differentiation. Immunity. (2014) 40:594–607. doi: 10.1016/j.immuni.2014.03.005 24684957 PMC4084624

[77] Dasgupta S Erturk-Hasdemir D Ochoa-Reparaz J Reinecker HC Kasper DL. Plasmacytoid dendritic cells mediate anti-inflammatory responses to a gut commensal molecule *via* both innate and adaptive mechanisms. Cell Host Microbe. (2014) 15:413–23. doi: 10.1016/j.chom.2014.03.006 PMC4020153 24721570

[78] Reber SO Siebler PH Donner NC Morton JT Smith DG Kopelman JM. Immunization with a heat-killed preparation of the environmental bacterium mycobacterium vaccae promotes stress resilience in mice. Proc Natl Acad Sci United States America. (2016) 113:E3130–9. doi: 10.1073/pnas.1600324113 PMC4896712 27185913

[79] Koloski NA Jones M Kalantar J Weltman M Zaguirre J Talley NJ. The brain–gut pathway in functional gastrointestinal disorders is bidirectional: A 12-year prospective population-based study. Gut. (2012) 61:1284–90. doi: 10.1136/gutjnl-2011-300474 22234979

[80] Koloski NA Jones M Talley NJ. Evidence that independent gut-to-brain and brain-to-gut pathways operate in the irritable bowel syndrome and functional dyspepsia: A 1-year population-based prospective study. Alimentary Pharmacol Ther. (2016) 44:592–600. doi: 10.1111/apt.13738 27444264

[81] Goodhand JR Kamperidis N Sirwan B Macken L Tshuma N Koodun Y. Factors associated with thiopurine non-adherence in patients with inflammatory bowel disease. Alimentary Pharmacol Ther. (2013) 38:1097–108. doi: 10.1111/apt.12476 24099471

[82] Thomson CA McColl A Cavanagh J Graham GJ. Peripheral inflammation is associated with remote global gene expression changes in the brain. J Neuroinflamm. (2014) 11:73. doi: 10.1186/1742-2094-11-73 PMC4022192 24708794

[83] D'Mello C Swain MG. Immune-to-brain communication pathways in inflammation-associated sickness and depression. Curr topics Behav Neurosci. (2017) 31:73–94. doi: 10.1007/7854_2016_37 27677781

[84] Steinberg BE Silverman HA Robbiati S Gunasekaran MK Tsaava T Battinelli E. Cytokine-specific neurograms in the sensory vagus nerve. Bioelectronic Med. (2016) 3:7–17. PMC6039192 30003120

[85] Watkins LR Goehler LE Relton JK Tartaglia N Silbert L Martin D. Blockade of interleukin-1 induced hyperthermia by subdiaphragmatic vagotomy: evidence for vagal mediation of immune-brain communication. Neurosci Lett. (1995) 183:27–31. doi: 10.1016/0304-3940(94)11105-r 7746479

[86] Wang FB Powley TL. Vagal innervation of intestines: afferent pathways mapped with new en bloc horseradish peroxidase adaptation. Cell Tissue Res. (2007) 329:221–30. doi: 10.1007/s00441-007-0413-7 17453246

[87] Bonaz B Bazin T Pellissier S. The vagus nerve at the interface of the microbiota-gut-brain axis. Front Neurosci. (2018) 12:49. doi: 10.3389/fnins.2018.00049 29467611 PMC5808284

[88] Koh A De Vadder F Kovatcheva-Datchary P Bäckhed F. From dietary fiber to host physiology: short-chain fatty acids as key bacterial metabolites. Cell. (2016) 165:1332–45. doi: 10.1016/j.cell.2016.05.041 27259147

[89] Raybould HE. Gut chemosensing: interactions between gut endocrine cells and visceral afferents. Auton Neurosci. (2010) 153:41–6. doi: 10.1016/j.autneu.2009.07.007 PMC3014315 19674941

[90] Tanida M Yamano T Maeda K Okumura N Fukushima Y Nagai K. Effects of intraduodenal injection of lactobacillus johnsonii la1 on renal sympathetic nerve activity and blood pressure in urethane-anesthetized rats. Neurosci Lett. (2005) 389:109–14. doi: 10.1016/j.neulet.2005.07.036 16118039

[91] Marcondes Ávila PR Fiorot M Michels M Dominguini D Abatti M Vieira A. Effects of microbiota transplantation and the role of the vagus nerve in gut-brain axis in animals subjected to chronic mild stress. J Affect Disord. (2020) 277:410–6. doi: 10.1016/j.jad.2020.08.013 32866799

[92] Haj-Mirzaian A Amiri S Amini-Khoei H Hosseini MJ Haj-Mirzaian A Momeny M. Anxiety- and depressive-like behaviors are associated with altered hippocampal energy and inflammatory status in a mouse model of crohn's disease. Neuroscience. (2017) 366:124–37. doi: 10.1016/j.neuroscience.2017.10.023 29080717

[93] Heydarpour P Rahimian R Fakhfouri G Khoshkish S Fakhraei N Salehi-Sadaghiani M. Behavioral despair associated with a mouse model of crohn's disease: role of nitric oxide pathway. Prog Neuropsychopharmacol Biol Psychiatry. (2016) 64:131–41. doi: 10.1016/j.pnpbp.2015.08.004 26268932

[94] Riazi K Galic MA Kuzmiski JB Ho W Sharkey KA Pittman QJ. Microglial activation and tnfalpha production mediate altered cns excitability following peripheral inflammation. Proc Natl Acad Sci United States America. (2008) 105:17151–6. doi: 10.1073/pnas.0806682105 PMC2579393 18955701

[95] Sierra A Encinas JM Deudero JJ Chancey JH Enikolopov G Overstreet-Wadiche LS. Microglia shape adult hippocampal neurogenesis through apoptosis-coupled phagocytosis. Cell Stem Cell. (2010) 7:483–95. doi: 10.1016/j.stem.2010.08.014 PMC4008496 20887954

[96] Takahashi K Kurokawa K Miyagawa K Mochida-Saito A Nemoto Y Iwasa H. Antidementia effects of enterococcus faecalis 2001 are associated with enhancement of hippocampal neurogenesis *via* the erk-creb-bdnf pathway in olfactory bulbectomized mice. Physiol Behav. (2020) 223:112997. doi: 10.1016/j.physbeh.2020.112997 32502526

[97] Gampierakis IA Koutmani Y Semitekolou M Morianos I Polissidis A Katsouda A. Hippocampal neural stem cells and microglia response to experimental inflammatory bowel disease (Ibd). Mol Psychiatry. (2021) 26:1248–63. doi: 10.1038/s41380-020-0651-6 31969694

[98] Zonis S Pechnick RN Ljubimov VA Mahgerefteh M Wawrowsky K Michelsen KS. Chronic intestinal inflammation alters hippocampal neurogenesis. J Neuroinflamm. (2015) 12:65. doi: 10.1186/s12974-015-0281-0 PMC4403851 25889852

[99] Liang S Wang T Hu X Luo J Li W Wu X. Administration of lactobacillus helveticus ns8 improves behavioral, cognitive, and biochemical aberrations caused by chronic restraint stress. Neuroscience. (2015) 310:561–77. doi: 10.1016/j.neuroscience.2015.09.033 26408987

[100] Wang T Hu X Liang S Li W Wu X Wang L. Lactobacillus fermentum ns9 restores the antibiotic induced physiological and psychological abnormalities in rats. Beneficial Microbes. (2015) 6:707–17. doi: 10.3920/bm2014.0177 25869281

[101] Tian P Wang G Zhao J Zhang H Chen W. Bifidobacterium with the role of 5-hydroxytryptophan synthesis regulation alleviates the symptom of depression and related microbiota dysbiosis. J Nutr Biochem. (2019) 66:43–51. doi: 10.1016/j.jnutbio.2019.01.007 30743155

[102] Tian P O'Riordan KJ Lee YK Wang G Zhao J Zhang H. Towards a psychobiotic therapy for depression: bifidobacterium breve ccfm1025 reverses chronic stress-induced depressive symptoms and gut microbial abnormalities in mice. Neurobiol Stress. (2020) 12:100216. doi: 10.1016/j.ynstr.2020.100216 32258258 PMC7109524

[103] Rao J Qiao Y Xie R Lin L Jiang J Wang C. Fecal microbiota transplantation ameliorates stress-induced depression-like behaviors associated with the inhibition of glial and nlrp3 inflammasome in rat brain. J Psychiatr Res. (2021) 137:147–57. doi: 10.1016/j.jpsychires.2021.02.057 33677218

[104] Bernstein CN Singh S Graff LA Walker JR Miller N Cheang M. A prospective population-based study of triggers of symptomatic flares in ibd. Am J Gastroenterol. (2010) 105:1994–2002. doi: 10.1038/ajg.2010.140 20372115

[105] Zegarra-Ruiz DF Kim DV Norwood K Kim M Wu WH Saldana-Morales FB. Thymic development of gut-microbiota-specific T cells. Nature. (2021) 594:413–7. doi: 10.1038/s41586-021-03531-1 PMC8323488 33981034

[106] Ivanov II Atarashi K Manel N Brodie EL Shima T Karaoz U. Induction of intestinal th17 cells by segmented filamentous bacteria. Cell. (2009) 139:485–98. doi: 10.1016/j.cell.2009.09.033 PMC2796826 19836068

[107] Lavelle A Sokol H. Gut microbiota-derived metabolites as key actors in inflammatory bowel disease. Nat Rev Gastroenterol Hepatol. (2020) 17:223–37. doi: 10.1038/s41575-019-0258-z 32076145

[108] Huda-Faujan N Abdulamir AS Fatimah AB Anas OM Shuhaimi M Yazid AM. The impact of the level of the intestinal short chain fatty acids in inflammatory bowel disease patients versus healthy subjects. Open Biochem J. (2010) 4:53–8. doi: 10.2174/1874091x01004010053 PMC2887640 20563285

[109] Stolzer I Kaden-Volynets V Ruder B Letizia M Bittel M Rausch P. Environmental microbial factors determine the pattern of inflammatory lesions in a murine model of crohn's disease-like inflammation. Inflammatory bowel Dis. (2020) 26:66–79. doi: 10.1093/ibd/izz142 31276162

[110] Yuan X Chen B Duan Z Xia Z Ding Y Chen T. Depression and anxiety in patients with active ulcerative colitis: crosstalk of gut microbiota, metabolomics and proteomics. Gut Microbes. (2021) 13:1987779. doi: 10.1080/19490976.2021.1987779 34806521 PMC8632339

[111] Klawonn AM Fritz M Castany S Pignatelli M Canal C Similä F. Microglial activation elicits a negative affective state through prostaglandin-mediated modulation of striatal neurons. Immunity. (2021) 54:225–34.e6. doi: 10.1016/j.immuni.2020.12.016 33476547

[112] Cao P Chen C Liu A Shan Q Zhu X Jia C. Early-life inflammation promotes depressive symptoms in adolescence *via* microglial engulfment of dendritic spines. Neuron. (2021) 109:2573–89.e9. doi: 10.1016/j.neuron.2021.06.012 34233151

[113] Ji C Tang Y Zhang Y Li C Liang H Ding L. Microglial glutaminase 1 deficiency mitigates neuroinflammation associated depression. Brain behavior Immun. (2022) 99:231–45. doi: 10.1016/j.bbi.2021.10.009 34678461

[114] Dang R Wang M Li X Wang H Liu L Wu Q. Edaravone ameliorates depressive and anxiety-like behaviors *via* sirt1/nrf2/ho-1/gpx4 pathway. J Neuroinflamm. (2022) 19:41. doi: 10.1186/s12974-022-02400-6 PMC8822843 35130906

[115] Chen Y Colonna M. Microglia in alzheimer's disease at single-cell level. Are there common patterns in humans and mice? J Exp Med. (2021) 218:e20202717. doi: 10.1084/jem.20202717 34292312 PMC8302448

[116] Crider A Feng T Pandya CD Davis T Nair A Ahmed AO. Complement component 3a receptor deficiency attenuates chronic stress-induced monocyte infiltration and depressive-like behavior. Brain behavior Immun. (2018) 70:246–56. doi: 10.1016/j.bbi.2018.03.004 PMC5967612 29518530

[117] Park H Poo MM. Neurotrophin regulation of neural circuit development and function. Nat Rev Neurosci. (2013) 14:7–23. doi: 10.1038/nrn3379 23254191

[118] Li W Ali T He K Liu Z Shah FA Ren Q. Ibrutinib alleviates lps-induced neuroinflammation and synaptic defects in a mouse model of depression. Brain behavior Immun. (2021) 92:10–24. doi: 10.1016/j.bbi.2020.11.008 33181270

[119] Barrientos RM Sprunger DB Campeau S Higgins EA Watkins LR Rudy JW. Brain-derived neurotrophic factor mrna downregulation produced by social isolation is blocked by intrahippocampal interleukin-1 receptor antagonist. Neuroscience. (2003) 121:847–53. doi: 10.1016/s0306-4522(03)00564-5 14580934

[120] Talley S Valiauga R Anderson L Cannon AR Choudhry MA Campbell EM. Dss-induced inflammation in the colon drives a proinflammatory signature in the brain that is ameliorated by prophylactic treatment with the S100a9 inhibitor paquinimod. J Neuroinflamm. (2021) 18:263. doi: 10.1186/s12974-021-02317-6 PMC8578918 34758843

[121] Nakagawasai O Yamada K Takahashi K Odaira T Sakuma W Ishizawa D. Liver hydrolysate prevents depressive-like behavior in an animal model of colitis: involvement of hippocampal neurogenesis *via* the ampk/bdnf pathway. Behav Brain Res. (2020) 390:112640. doi: 10.1016/j.bbr.2020.112640 32434062

[122] Erny D Hrabě de Angelis AL Jaitin D Wieghofer P Staszewski O David E. Host microbiota constantly control maturation and function of microglia in the cns. Nat Neurosci. (2015) 18:965–77. doi: 10.1038/nn.4030 PMC5528863 26030851

[123] Duscha A Gisevius B Hirschberg S Yissachar N Stangl GI Dawin E. Propionic acid shapes the multiple sclerosis disease course by an immunomodulatory mechanism. Cell. (2020) 180:1067–80.e16. doi: 10.1016/j.cell.2020.02.035 32160527

[124] Rothhammer V Borucki DM Tjon EC Takenaka MC Chao CC Ardura-Fabregat A. Microglial control of astrocytes in response to microbial metabolites. Nature. (2018) 557:724–8. doi: 10.1038/s41586-018-0119-x PMC6422159 29769726

[125] Cryan JF Dinan TG. Mind-altering microorganisms: the impact of the gut microbiota on brain and behaviour. Nat Rev Neurosci. (2012) 13:701–12. doi: 10.1038/nrn3346 22968153

[126] Bercik P Verdu EF Foster JA Macri J Potter M Huang X. Chronic gastrointestinal inflammation induces anxiety-like behavior and alters central nervous system biochemistry in mice. Gastroenterology. (2010) 139:2102–12.e1. doi: 10.1053/j.gastro.2010.06.063 20600016

[127] Barrett E Ross RP O'Toole PW Fitzgerald GF Stanton C. Γ-aminobutyric acid production by culturable bacteria from the human intestine. J Appl Microbiol. (2012) 113:411–7. doi: 10.1111/j.1365-2672.2012.05344.x 22612585

[128] Bravo JA Forsythe P Chew MV Escaravage E Savignac HM Dinan TG. Ingestion of lactobacillus strain regulates emotional behavior and central gaba receptor expression in a mouse *via* the vagus nerve. Proc Natl Acad Sci United States America. (2011) 108:16050–5. doi: 10.1073/pnas.1102999108 PMC3179073 21876150

[129] Zhang Y Fan Q Hou Y Zhang X Yin Z Cai X. Bacteroides species differentially modulate depression-like behavior *via* gut-brain metabolic signaling. Brain behavior Immun. (2022) 102:11–22. doi: 10.1016/j.bbi.2022.02.007 35143877

[130] Huang W Jiang SM Jia L You LQ Huang YX Gong YM. Effect of amitriptyline on gastrointestinal function and brain-gut peptides: A double-blind trial. World J Gastroenterol. (2013) 19:4214–20. doi: 10.3748/wjg.v19.i26.4214 PMC3710425 23864786

[131] Tynan RJ Weidenhofer J Hinwood M Cairns MJ Day TA Walker FR. A comparative examination of the anti-inflammatory effects of ssri and snri antidepressants on lps stimulated microglia. Brain behavior Immun. (2012) 26:469–79. doi: 10.1016/j.bbi.2011.12.011 22251606

[132] Ha E Jung KH Choe BK Bae JH Shin DH Yim SV. Fluoxetine increases the nitric oxide production via nuclear factor kappa B-mediated pathway in bv2 murine microglial cells. Neurosci Lett. (2006) 397:185–9. doi: 10.1016/j.neulet.2005.12.022 16413968

[133] Branco-de-Almeida LS Kajiya M Cardoso CR Silva MJ Ohta K Rosalen PL. Selective serotonin reuptake inhibitors attenuate the antigen presentation from dendritic cells to effector T lymphocytes. FEMS Immunol Med Microbiol. (2011) 62:283–94. doi: 10.1111/j.1574-695X.2011.00816.x PMC3147168 21569123

[134] Bannister K Patel R Goncalves L Townson L Dickenson AH. Diffuse noxious inhibitory controls and nerve injury: restoring an imbalance between descending monoamine inhibitions and facilitations. Pain. (2015) 156:1803–11. doi: 10.1097/j.pain.0000000000000240 26010460

[135] Nakajima K Obata H Iriuchijima N Saito S. An increase in spinal cord noradrenaline is a major contributor to the antihyperalgesic effect of antidepressants after peripheral nerve injury in the rat. Pain. (2012) 153:990–7. doi: 10.1016/j.pain.2012.01.029 22424692

[136] Carvalho AF Sharma MS Brunoni AR Vieta E Fava GA. The safety, tolerability and risks associated with the use of newer generation antidepressant drugs: A critical review of the literature. Psychother psychosomatics. (2016) 85:270–88. doi: 10.1159/000447034 27508501

[137] El Mansari M Ghanbari R Janssen S Blier P. Sustained administration of bupropion alters the neuronal activity of serotonin, norepinephrine but not dopamine neurons in the rat brain. Neuropharmacology. (2008) 55:1191–8. doi: 10.1016/j.neuropharm.2008.07.028 18708076

[138] Kim JK Han SK Joo MK Kim DH. Buspirone alleviates anxiety, depression, and colitis; and modulates gut microbiota in mice. Sci Rep. (2021) 11:6094. doi: 10.1038/s41598-021-85681-w 33731795 PMC7969772

[139] Gershon MD Tack J. The serotonin signaling system: from basic understanding to drug development for functional gi disorders. Gastroenterology. (2007) 132:397–414. doi: 10.1053/j.gastro.2006.11.002 17241888

[140] Tracey I Mantyh PW. The cerebral signature for pain perception and its modulation. Neuron. (2007) 55:377–91. doi: 10.1016/j.neuron.2007.07.012 17678852

[141] Brunoni AR Lopes M Fregni F. A systematic review and meta-analysis of clinical studies on major depression and bdnf levels: implications for the role of neuroplasticity in depression. Int J Neuropsychopharmacol. (2008) 11:1169–80. doi: 10.1017/s1461145708009309 18752720

[142] Geddes JR Carney SM Davies C Furukawa TA Kupfer DJ Frank E. Relapse prevention with antidepressant drug treatment in depressive disorders: A systematic review. Lancet (London England). (2003) 361:653–61. doi: 10.1016/s0140-6736(03)12599-8 12606176

[143] Hall BJ Hamlin PJ Gracie DJ Ford AC. The effect of antidepressants on the course of inflammatory bowel disease. Can J Gastroenterol Hepatol. (2018) 2018:2047242. doi: 10.1155/2018/2047242 30271765 PMC6151237

[144] Goodhand JR Greig FI Koodun Y McDermott A Wahed M Langmead L. Do antidepressants influence the disease course in inflammatory bowel disease? A retrospective case-matched observational study. Inflammatory bowel Dis. (2012) 18:1232–9. doi: 10.1002/ibd.21846 22234954

[145] Takahashi K Nakagawasai O Nemoto W Odaira T Sakuma W Onogi H. Effect of enterococcus faecalis 2001 on colitis and depressive-like behavior in dextran sulfate sodium-treated mice: involvement of the brain-gut axis. J Neuroinflamm. (2019) 16:201. doi: 10.1186/s12974-019-1580-7 PMC6822456 31672153

[146] Emge JR Huynh K Miller EN Kaur M Reardon C Barrett KE. Modulation of the microbiota-gut-brain axis by probiotics in a murine model of inflammatory bowel disease. Am J Physiol Gastrointestinal liver Physiol. (2016) 310:G989–98. doi: 10.1152/ajpgi.00086.2016 27056723

[147] Sokol H Pigneur B Watterlot L Lakhdari O Bermúdez-Humarán LG Gratadoux JJ. Faecalibacterium prausnitzii is an anti-inflammatory commensal bacterium identified by gut microbiota analysis of crohn disease patients. Proc Natl Acad Sci United States America. (2008) 105:16731–6. doi: 10.1073/pnas.0804812105 PMC2575488 18936492

[148] Prosberg M Bendtsen F Vind I Petersen AM Gluud LL. The association between the gut microbiota and the inflammatory bowel disease activity: A systematic review and meta-analysis. Scandinavian J Gastroenterol. (2016) 51:1407–15. doi: 10.1080/00365521.2016.1216587 27687331

[149] Hao Z Wang W Guo R Liu H. Faecalibacterium prausnitzii (Atcc 27766) has preventive and therapeutic effects on chronic unpredictable mild stress-induced depression-like and anxiety-like behavior in rats. Psychoneuroendocrinology. (2019) 104:132–42. doi: 10.1016/j.psyneuen.2019.02.025 30844607

[150] Burokas A Arboleya S Moloney RD Peterson VL Murphy K Clarke G. Targeting the microbiota-gut-brain axis: prebiotics have anxiolytic and antidepressant-like effects and reverse the impact of chronic stress in mice. Biol Psychiatry. (2017) 82:472–87. doi: 10.1016/j.biopsych.2016.12.031 28242013

[151] Garcia Vilela E De Lourdes De Abreu Ferrari M Oswaldo Da Gama Torres H Guerra Pinto A Carolina Carneiro Aguirre A Paiva Martins F. Influence of saccharomyces boulardii on the intestinal permeability of patients with crohn's disease in remission. Scandinavian J Gastroenterol. (2008) 43:842–8. doi: 10.1080/00365520801943354 18584523

[152] Kato K Mizuno S Umesaki Y Ishii Y Sugitani M Imaoka A. Randomized placebo-controlled trial assessing the effect of bifidobacteria-fermented milk on active ulcerative colitis. Alimentary Pharmacol Ther. (2004) 20:1133–41. doi: 10.1111/j.1365-2036.2004.02268.x 15569116

[153] Groeger D O'Mahony L Murphy EF Bourke JF Dinan TG Kiely B. Bifidobacterium infantis 35624 modulates host inflammatory processes beyond the gut. Gut Microbes. (2013) 4:325–39. doi: 10.4161/gmic.25487 PMC3744517 23842110

[154] Tamaki H Nakase H Inoue S Kawanami C Itani T Ohana M. Efficacy of probiotic treatment with bifidobacterium longum 536 for induction of remission in active ulcerative colitis: A randomized, double-blinded, placebo-controlled multicenter trial. Dig Endosc. (2016) 28:67–74. doi: 10.1111/den.12553 26418574

[155] Shen M Shi Y Ge Z Qian J. Effects of mesalamine combined with live combined bifidobacterium, lactobacillus and enterococcus capsules on intestinal mucosa barrier function and intestinal microbiota in mildly active crohn's disease patients. J Invest Surg. (2024) 37:2297565. doi: 10.1080/08941939.2023.2297565 38159563

[156] Costello SP Hughes PA Waters O Bryant RV Vincent AD Blatchford P. Effect of fecal microbiota transplantation on 8-week remission in patients with ulcerative colitis: A randomized clinical trial. Jama. (2019) 321:156–64. doi: 10.1001/jama.2018.20046 PMC6439766 30644982

[157] Paramsothy S Kamm MA Kaakoush NO Walsh AJ van den Bogaerde J Samuel D. Multidonor intensive faecal microbiota transplantation for active ulcerative colitis: A randomised placebo-controlled trial. Lancet. (2017) 389:1218–28. doi: 10.1016/s0140-6736(17)30182-4 28214091

[158] Moayyedi P Surette MG Kim PT Libertucci J Wolfe M Onischi C. Fecal microbiota transplantation induces remission in patients with active ulcerative colitis in a randomized controlled trial. Gastroenterology. (2015) 149:102–9.e6. doi: 10.1053/j.gastro.2015.04.001 25857665

[159] Rossen NG Fuentes S van der Spek MJ Tijssen JG Hartman JH Duflou A. Findings from a randomized controlled trial of fecal transplantation for patients with ulcerative colitis. Gastroenterology. (2015) 149:110–8.e4. doi: 10.1053/j.gastro.2015.03.045 25836986

[160] Sood A Mahajan R Singh A Midha V Mehta V Narang V. Role of faecal microbiota transplantation for maintenance of remission in patients with ulcerative colitis: A pilot study. J Crohns Colitis. (2019) 13:1311–7. doi: 10.1093/ecco-jcc/jjz060 30873549

[161] Derwa Y Gracie DJ Hamlin PJ Ford AC. Systematic review with meta-analysis: the efficacy of probiotics in inflammatory bowel disease. Alimentary Pharmacol Ther. (2017) 46:389–400. doi: 10.1111/apt.14203 28653751

[162] Ng QX Peters C Ho CYX Lim DY Yeo WS. A meta-analysis of the use of probiotics to alleviate depressive symptoms. J Affect Disord. (2018) 228:13–9. doi: 10.1016/j.jad.2017.11.063 29197739

[163] Romijn AR Rucklidge JJ Kuijer RG Frampton C. A double-blind, randomized, placebo-controlled trial of lactobacillus helveticus and bifidobacterium longum for the symptoms of depression. Aust New Z J Psychiatry. (2017) 51:810–21. doi: 10.1177/0004867416686694 PMC5518919 28068788

[164] Evrensel A Ceylan ME. Fecal microbiota transplantation and its usage in neuropsychiatric disorders. Clin Psychopharmacol Neurosci. (2016) 14:231–7. doi: 10.9758/cpn.2016.14.3.231 PMC4977816 27489376

[165] Allegretti JR Mullish BH Kelly C Fischer M. The evolution of the use of faecal microbiota transplantation and emerging therapeutic indications. Lancet (London England). (2019) 394:420–31. doi: 10.1016/s0140-6736(19)31266-8 31379333

[166] Narula N Kassam Z Yuan Y Colombel JF Ponsioen C Reinisch W. Systematic review and meta-analysis: fecal microbiota transplantation for treatment of active ulcerative colitis. Inflammatory bowel Dis. (2017) 23:1702–9. doi: 10.1097/mib.0000000000001228 28906291

[167] De Palma G Lynch MD Lu J Dang VT Deng Y Jury J. Transplantation of fecal microbiota from patients with irritable bowel syndrome alters gut function and behavior in recipient mice. Sci Trans Med. (2017) 9:eaaf6397. doi: 10.1126/scitranslmed.aaf6397 28251905

[168] Graff LA Walker JR Clara I Lix L Miller N Rogala L. Stress coping, distress, and health perceptions in inflammatory bowel disease and community controls. Am J Gastroenterol. (2009) 104:2959–69. doi: 10.1038/ajg.2009.529 19755973

[169] Guthrie E Jackson J Shaffer J Thompson D Tomenson B Creed F. Psychological disorder and severity of inflammatory bowel disease predict health-related quality of life in ulcerative colitis and crohn's disease. Am J Gastroenterol. (2002) 97:1994–9. doi: 10.1111/j.1572-0241.2002.05842.x 12190166

[170] Kiebles JL Doerfler B Keefer L. Preliminary evidence supporting a framework of psychological adjustment to inflammatory bowel disease. Inflammatory bowel Dis. (2010) 16:1685–95. doi: 10.1002/ibd.21215 PMC2946432 20155849

[171] Keefer L Ballou SK Drossman DA Ringstrom G Elsenbruch S Ljótsson B. A rome working team report on brain-gut behavior therapies for disorders of gut-brain interaction. Gastroenterology. (2022) 162:300–15. doi: 10.1053/j.gastro.2021.09.015 34529986

[172] Zia JK Barney P Cain KC Jarrett ME Heitkemper MM. A comprehensive self-management irritable bowel syndrome program produces sustainable changes in behavior after 1 year. Clin Gastroenterol Hepatol. (2016) 14:212–9.e1-2. doi: 10.1016/j.cgh.2015.09.027 26453951 PMC4718771

[173] Hasan SS Pearson JS Morris J Whorwell PJ. Skype hypnotherapy for irritable bowel syndrome: effectiveness and comparison with face-to-face treatment. Int J Clin Exp hypnosis. (2019) 67:69–80. doi: 10.1080/00207144.2019.1553766 PMC6538308 30702396

[174] Peter J Fournier C Keip B Rittershaus N Stephanou-Rieser N Durdevic M. Intestinal Microbiome in Irritable Bowel Syndrome before and after Gut-Directed Hypnotherapy. Int J Mol Sci. (2018) 19:3619. doi: 10.3390/ijms19113619 30453528 PMC6274728

[175] Liegl G Plessen CY Leitner A Boeckle M Pieh C. Guided self-help interventions for irritable bowel syndrome: A systematic review and meta-analysis. Eur J Gastroenterol Hepatol. (2015) 27:1209–21. doi: 10.1097/meg.0000000000000428 26164395

[176] Hyphantis T Guthrie E Tomenson B Creed F. Psychodynamic interpersonal therapy and improvement in interpersonal difficulties in people with severe irritable bowel syndrome. Pain. (2009) 145:196–203. doi: 10.1016/j.pain.2009.07.005 19643544

[177] Drossman DA Ruddy J. Improving patient-provider relationships to improve health care. Clin Gastroenterol Hepatol. (2020) 18:1417–26. doi: 10.1016/j.cgh.2019.12.007 31843593

[178] Khalili H Chan SSM Lochhead P Ananthakrishnan AN Hart AR Chan AT. The role of diet in the aetiopathogenesis of inflammatory bowel disease. Nat Rev Gastroenterol Hepatol. (2018) 15:525–35. doi: 10.1038/s41575-018-0022-9 PMC6397648 29789682

[179] Charpentier C Chan R Salameh E Mbodji K Ueno A Coëffier M. Dietary N-3 pufa may attenuate experimental colitis. Mediators Inflammation. (2018) 2018:8430614. doi: 10.1155/2018/8430614 PMC5833476 29670469

[180] Costantini L Molinari R Farinon B Merendino N. Impact of omega-3 fatty acids on the gut microbiota. Int J Mol Sci. (2017) 18:2645. doi: 10.3390/ijms18122645 29215589 PMC5751248

[181] Robertson RC Seira Oriach C Murphy K Moloney GM Cryan JF Dinan TG. Omega-3 polyunsaturated fatty acids critically regulate behaviour and gut microbiota development in adolescence and adulthood. Brain behavior Immun. (2017) 59:21–37. doi: 10.1016/j.bbi.2016.07.145 27423492

[182] Pusceddu MM Kelly P Ariffin N Cryan JF Clarke G Dinan TG. N-3 pufas have beneficial effects on anxiety and cognition in female rats: effects of early life stress. Psychoneuroendocrinology. (2015) 58:79–90. doi: 10.1016/j.psyneuen.2015.04.015 25965872

[183] Hookway C Buckner S Crosland P Longson D. Irritable bowel syndrome in adults in primary care: summary of updated nice guidance. BMJ (Clinical Res ed). (2015) 350:h701. doi: 10.1136/bmj.h701 25716701

[184] Major G Pritchard S Murray K Alappadan JP Hoad CL Marciani L. Colon hypersensitivity to distension, rather than excessive gas production, produces carbohydrate-related symptoms in individuals with irritable bowel syndrome. Gastroenterology. (2017) 152:124–33.e2. doi: 10.1053/j.gastro.2016.09.062 27746233

[185] Cox SR Lindsay JO Fromentin S Stagg AJ McCarthy NE Galleron N. Effects of low fodmap diet on symptoms, fecal microbiome, and markers of inflammation in patients with quiescent inflammatory bowel disease in a randomized trial. Gastroenterology. (2020) 158:176–88.e7. doi: 10.1053/j.gastro.2019.09.024 31586453

[186] Halmos EP Christophersen CT Bird AR Shepherd SJ Gibson PR Muir JG. Diets that differ in their fodmap content alter the colonic luminal microenvironment. Gut. (2015) 64:93–100. doi: 10.1136/gutjnl-2014-307264 25016597

[187] van Langenberg DR Gibson PR. Factors associated with physical and cognitive fatigue in patients with crohn's disease: A cross-sectional and longitudinal study. Inflammatory bowel Dis. (2014) 20:115–25. doi: 10.1097/01.Mib.0000437614.91258.70 24297056

[188] Lo CH Khalili H Song M Lochhead P Burke KE Richter JM. Healthy lifestyle is associated with reduced mortality in patients with inflammatory bowel diseases. Clin Gastroenterol Hepatol. (2021) 19:87–95.e4. doi: 10.1016/j.cgh.2020.02.047 32142939 PMC7483199

[189] Torres J Ellul P Langhorst J Mikocka-Walus A Barreiro-de Acosta M Basnayake C. European crohn's and colitis organisation topical review on complementary medicine and psychotherapy in inflammatory bowel disease. J Crohn's colitis. (2019) 13:673–85e. doi: 10.1093/ecco-jcc/jjz051 30820529

[190] Gatt K Schembri J Katsanos KH Christodoulou D Karmiris K Kopylov U. Inflammatory bowel disease [Ibd] and physical activity: A study on the impact of diagnosis on the level of exercise amongst patients with ibd. J Crohn's colitis. (2019) 13:686–92. doi: 10.1093/ecco-jcc/jjy214 30561568

[191] Greenley RN Naftaly JP Walker RJ Kappelman MD Martin CF Schneider KL. Sports participation in youth with inflammatory bowel diseases: the role of disease activity and subjective physical health symptoms. Inflammatory bowel Dis. (2018) 24:247–53. doi: 10.1093/ibd/izx057 29361104

[192] Jain A Nguyen NH Proudfoot JA Martin CF Sandborn WJ Kappelman MD. Impact of obesity on disease activity and patient-reported outcomes measurement information system (Promis) in inflammatory bowel diseases. Am J Gastroenterol. (2019) 114:630–9. doi: 10.14309/ajg.0000000000000197 PMC6824268 30865012

[193] Nguyen NH Ohno-MaChado L Sandborn WJ Singh S. Obesity is independently associated with higher annual burden and costs of hospitalization in patients with inflammatory bowel diseases. Clin Gastroenterol Hepatol. (2019) 17:709–18.e7. doi: 10.1016/j.cgh.2018.07.004 30012429

[194] Lou H Liu X Liu P. Mechanism and implications of pro-nature physical activity in antagonizing psychological stress: the key role of microbial-gut-brain axis. Front Psychol. (2023) 14:1143827. doi: 10.3389/fpsyg.2023.1143827 37560094 PMC10408457

[195] Ulmer JM Wolf KL Backman DR Tretheway RL Blain CJ O'Neil-Dunne JP. Multiple health benefits of urban tree canopy: the mounting evidence for a green prescription. Health place. (2016) 42:54–62. doi: 10.1016/j.healthplace.2016.08.011 27639106

[196] Tzemah Shahar R Koren O Matarasso S Shochat T Magzal F Agmon M. Attributes of physical activity and gut microbiome in adults: A systematic review. Int J sports Med. (2020) 41:801–14. doi: 10.1055/a-1157-9257 32455454

[197] DeFilippis EM Tabani S Warren RU Christos PJ Bosworth BP Scherl EJ. Exercise and self-reported limitations in patients with inflammatory bowel disease. Digestive Dis Sci. (2016) 61:215–20. doi: 10.1007/s10620-015-3832-4 26254773

[198] Holik D Včev A Milostić-Srb A Salinger Ž Ivanišević Z Včev I. The effect of daily physical activity on the activity of inflammatory bowel diseases in therapy-free patients. Acta clinica Croatica. (2019) 58:202–12. doi: 10.20471/acc.2019.58.02.02 PMC6884387 31819315

